# WhiFuN: A toolbox to map the white matter functional networks of the human brain

**DOI:** 10.1162/IMAG.a.3

**Published:** 2025-05-30

**Authors:** Pratik Jain, Andrew M. Michael, Pan Wang, Xin Di, Bharat Biswal

**Affiliations:** New Jersey Institute of Technology, Newark, NJ, United States; Rutgers School of Graduate Studies, Newark, NJ, United States; Duke Institute for Brain Sciences, Duke University, Durham, NC, United States; The Clinical Hospital of Chengdu Brain Science Institute, MOE Key Laboratory for Neuroinformation, Center for Information in Medicine, School of Life Science and Technology, University of Electronic Science and Technology of China, Chengdu, China

**Keywords:** white matter, functional connectivity, white matter fMRI, software toolbox

## Abstract

Functional connectivity (FC) computed using functional magnetic resonance imaging (fMRI) of the gray matter (GM) regions of the human brain has been successfully used to find reliable markers of healthy and clinical populations. Approximately 50% of the human brain consists of white matter (WM), and previous studies have shown the presence of blood oxygen level-dependent (BOLD) signals in the WM. However, current FC analysis by researchers is limited to GM regions of the brain, and fMRI data from WM are typically not analyzed. Here, we present the White Matter Functional Networks (WhiFuN) Toolbox specifically designed for WM-FC analysis, incorporating preprocessing steps that minimize signal contamination due to GM, optimized methods for extracting meaningful WM signals, and dedicated statistical and visualization tools for WM-FC. WhiFuN is based on SPM12 preprocessing and contains statistical tools for group-level analyses. WhiFuN provides an intuitive graphical user interface allowing users to execute all steps from preprocessing to final group-level analyses and does not require prior knowledge of computer programming. To demonstrate the features and capabilities of WhiFuN, 98 healthy controls from the publicly available HCP 100 unrelated dataset were used to identify sex differences in WM-FC. We found significant WM-FC sex differences between the left body of the corpus callosum (CC) and the WM-FN that included the left and right posterior corona radiata and the left and right posterior thalamic region. WhiFuN will provide a platform for the neuroimaging community, offering new dimensions to elucidate human brain function as an integrated system of both GM and WM.

## 1 Introduction

Functional magnetic resonance imaging (fMRI) signals acquired using the blood oxygen level-dependent (BOLD) paradigm have been widely used to provide information about the functional activation of the human brain. These fMRI BOLD signals are primarily analyzed from the brain’s gray matter (GM) regions. The white matter (WM) fMRI signals are typically ignored as WM’s BOLD signal amplitude is significantly lower than GM (Logothetis & Wandell, 2004). Consequently, many fMRI studies use the average WM signal as noise and regress it from every GM voxel time series to capture GM BOLD signal changes. A recent review byGrajauskas et al. (2019)has reported BOLD activation in the WM, emphasizing that the WM signal should not be used as a nuisance regressor. Although the physiological origins of WM BOLD signals are not entirely understood, it is currently believed that while the BOLD signal from the GM is associated with postsynaptic potentials, the fMRI BOLD signals from the WM are associated with action potentials (Gawryluk et al., 2014). Moreover, there is growing evidence that the WM BOLD signals are highly correlated with intracranial electrophysiological signals across a wide range of frequency bands (Huang et al., 2023).

Studies of task-based fMRI demonstrated activations in WM BOLD signals, suggesting that they are not random noise.Tettamanti et al. (2002)reported activation in the corpus callosum (CC) during a visuomotor task, which was replicated byWeber et al. (2005)and later extensively studied byGawryluk et al. (2009). Beyond the Corpus callosum, activations were observed in the other WM regions in response to tasks such as working memory, episodic memory, decision-making, and affective rating tasks (Yarkoni et al., 2009). Using Object and Face recognition tasks, BOLD time courses corresponding to WM tracts connected to bilateral fusiform gyrus have been shown to be modulated by a high-level visual processing task (M. Li, Ding, et al., 2020). There is also evidence that visual and motor tasks influence the correlations between WM and GM regions similar to cortical regions (Ding et al., 2018). In another study, It was observed that the power of the low-frequency BOLD fluctuations in the optical radiations WM tract was higher during visual tasks than during rest (Ji et al., 2017). Furthermore, changing the frequency of a flickering visual stimulus modulated the frequencies in the WM-BOLD signal in optical WM tracks (Mishra et al., 2020). During movie-watching, others have demonstrated the consistency in WM-functional networks (WM-FNs) created using both independent component analysis (ICA) (Marussich et al., 2017) and K-means clustering (Hu et al., 2023).

Several recent studies have analyzed WM BOLD signals in resting-state fMRI (rsfMRI) data sets. Researchers observed WM-FNs that were symmetric across both hemispheres created using K-means algorithm (Peer et al., 2017;P. Wang et al., 2020,^2022^).Peer et al. (2017)have also demonstrated that the WM-FNs are consistent and reproducible in a larger number (n = 176) of participants. They created WM-FNs from randomly selected subgroups of total participants. They used the Dice coefficient to quantify the similarity between the WM-FNs and computed their stability by increasing the number of randomly selected subgroups of participants. The WM-FNs found using n = 60 and n = 30 subgroups were similar to the clustering solution found using all the participants. Other studies have validated the WM-FNs using various approaches.Huang et al. (2020)demonstrated that symmetric WM-FNs are reproducible across different datasets, such as the Chinese human connectome project and human connectome project, using ICA. The above studies show that WM-FNs are stable and reliably reproducible across cohorts.

To further understand the connectivity between the different WM-FNs, WM-FC is computed by correlating the average fMRI time series extracted from FNs (E. A. Allen et al., 2011;Anderson & Cohen, 2013). The stability of the WM-FC was tested using fMRI runs acquired on 2 different days (P. Wang et al., 2020). The correlation between the WM-FC matrices across two runs was 0.995, and the correlation between the FC computed by cross-correlating the time series from WM-FNs and GM-FNs (WM-GM-FC) was 0.993, which further substantiates that the WM-FNs are not only spatially reproducible across groups of participants but also reproducible in terms of their FC patterns. Further, WM-FC computed from ICA-derived WM-FNs also showed distinct patterns of WM correlations reproducible across different datasets (Huang et al., 2020), proving that data-driven approaches can also identify WM-FNs reliably.

FC computed using the WM and GM-FNs created from K-means clustering have been used to find differences between patient populations and matched healthy controls.Jiang et al. (2019)found that the perception motor system is disrupted in patients with schizophrenia in the WM regions, suggesting that WM-FC analysis showed inefficient communication in schizophrenia. In another independent study of patients with schizophrenia, differences in corona radiata and cerebellum were found (X. Wang et al., 2021). WM-FC have been analyzed to identify differences in conduct disorder and healthy controls, and significant differences in the connections between the WM orbitofrontal network, corona radiata network occipital network, and superior temporal network were found (Lu et al., 2023).Sansone et al. (2023)found a significant overlap of the glioblastoma core and edema between the GM and WM-FNs. The connection between WM regions and the hippocampus was affected in patients with temporal lobe epilepsy (X. Li et al., 2022). Compared to healthy controls, a significantly higher FC was observed between the inferior and superior longitudinal fasciculus FNs in myotonic dystrophy patients (J. Li et al., 2022).Lin et al. (2021)demonstrated in their study that the mild cognitive impairment (MCI) APOE4 carriers showed decreased FC at the right temporopolaris, left corticospinal tract, and bilateral posterior limb of the internal capsule compared to MCI patients who did not have the APOE4 gene. These studies show that WM-FC is altered in patient populations, suggesting that analysis pipelines should include WM regions in addition to GM.

The BOLD signals in the WM have been shown to exhibit anisotropic temporal correlations with neighboring voxels, and these functional correlational tensors (FCT) are consistent with those revealed by diffusion tensors (Ding et al., 2013;Schilling et al., 2019). Similarly,Peer et al. (2017)observed that WM-FNs overlap with the structural tracts and that several FNs extend across several Diffusion Tensor Imaging (DTI) tracts and vice versa.Huang et al. (2020)showed an overlap between the ICA-derived WM-FNs and the JHU atlas created using DTI data (Mori et al., 2008).P. Wang et al. (2021)showed that there was considerable overlap between WM-FNs connected to the CC with that of the structural connection computed by DTI. Another study byDing et al. (2016)observed that different tasks can cause changes in the FCT and induce anisotropic correlations that were otherwise absent in the rsfMRI. This evidence suggests that the BOLD signal from WM may be driven by neural activity in the fiber tracts.

Given the recent studies on WM-FC and WM-FC differences between patient populations and healthy controls, researchers must incorporate WM-FC analysis in addition to the traditional GM-FC analyses. To help facilitate, we have developed the White Matter Functional Networks (WhiFuN) toolbox focused on preprocessing and analyzing the WM-FNs. WhiFuN was developed using MATLAB, incorporating widely used fMRI analysis algorithms to compute WM-FNs. WhiFuN provides a user-friendly Graphical User interface (GUI) interface to perform preprocessing steps and quality control analysis of different preprocessing stages, as previously suggested byDi and Biswal (2023). WhiFuN uses the voxel-level FC matrix as input to the K-means clustering algorithm to construct the WM-FNs and GM-FNs in a data-driven manner. It can extract average time series signals from the regions of interest (ROIs)/FNs and identify group differences between cohorts or associations with behavioral data with appropriate statistical tests. Users can also access standard WM atlases, such as the JHU atlas (Mori et al., 2008;Oishi et al., 2009), or manually provide a new atlas to define the ROIs and create the corresponding FC.

To demonstrate the application of WhiFuN, we present sex differences in the WM-FNs and GM-FNs extracted by K-means clustering applied to data from the human connectome project (HCP) 100 unrelated dataset (Van Essen et al., 2012). We used the resting-state scans corresponding to two sessions (rest1 LR and rest2 LR) from the HCP dataset to demonstrate the results.

## 2 Materials and Methods

WhiFuN was developed using MATLAB (version R2022b) under the Windows environment. The WhiFuN GUI was developed using MATLAB App Designer. In addition to custom-written code, several functions from SPM12 (https://www.fil.ion.ucl.ac.uk/spm/) were used for preprocessing and quality control. WhiFuN saves quality control plots to assess the quality of preprocessing steps. Users may select the data corresponding to participants (using the*uigetfile_n_dir*function developed byTiago (2024)) for which the preprocessing was not done as desired and discard them from further analysis. Visualization of the WM and GM-FNs was performed in conjunction with the BrainNet viewer toolbox (Xia et al., 2013).

WhiFuN expects every participant to have the anatomical (T1) and the functional scans (BOLD-rsfMRI) in the*Nifti*file format. It uses the Brain Imaging Data Structure (BIDS) to read the images from the participant folders. Nevertheless, non-BIDS formats are supported by specifying the folder structure and folder names corresponding to the desired dataset. After preprocessing, WhiFuN can be used to create the WM and the GM-FNs, which are stored in*Nifti*format in the WhiFuN output folder. The BrainNet viewer images from 6 different views (Anterior, Posterior, Dorsal, Ventral, left, and right) are stored for every WM and GM-FN as*.png*files. WhiFuN also extracts the average time series from every FN corresponding to every participant and stores it as a*.mat*file. The average time series corresponding to any other predefined atlas can also be stored as a*.mat*file using the toolbox. Furthermore, average time series filtered within specific frequency bands can be extracted and saved as*.mat*.

### 2.1 Features of WhiFuN

**Fig. 1.**
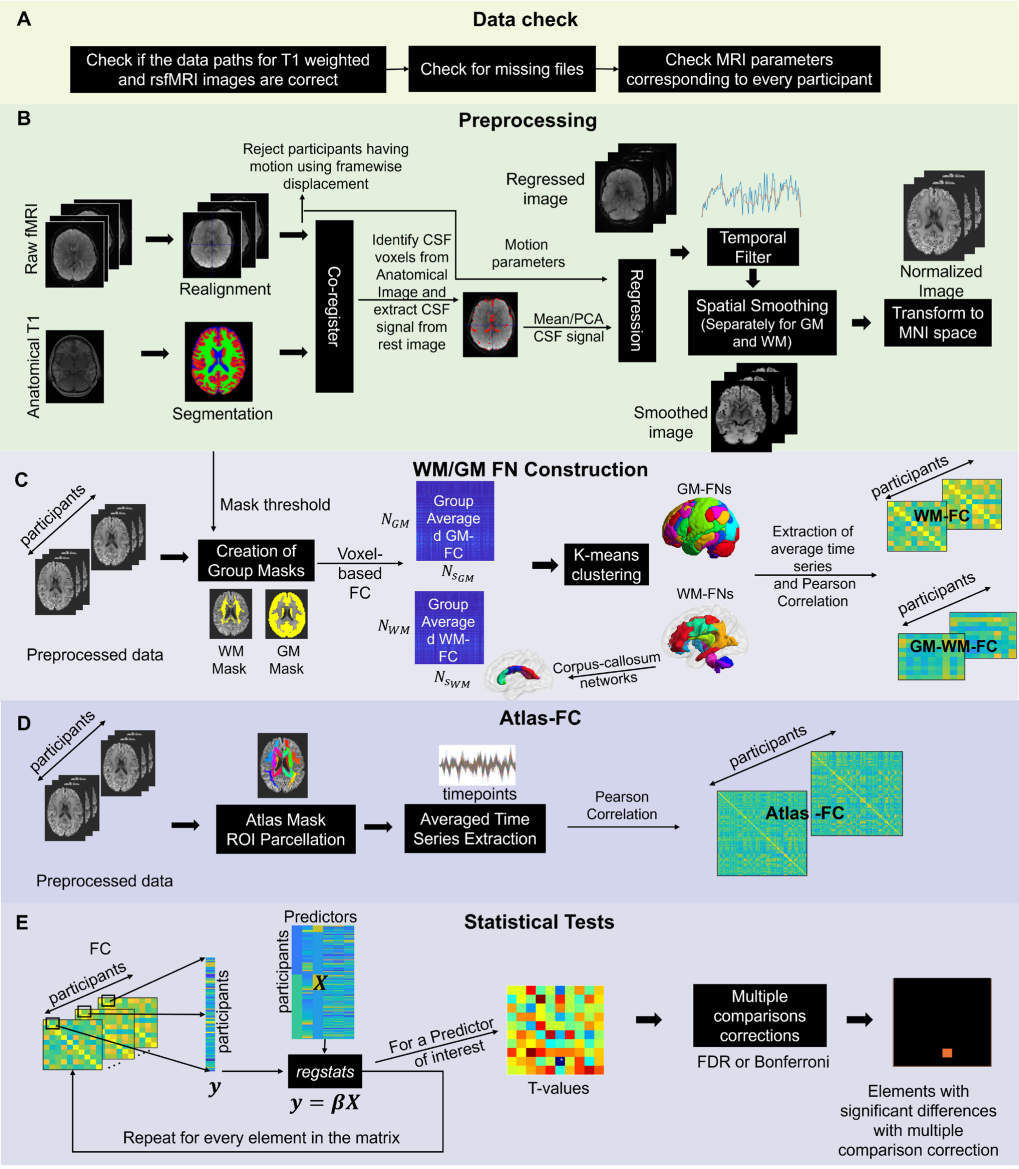
Schematic representation of WhiFuN. (A) Data Check: WhiFuN checks any missing files and consistency of MR parameters across all images. (B) Preprocessing: WhiFuN performs Realignment, Framewise displacement, segmentation of anatomical images, regression of noise temporal filtering, spatial smoothing, and normalization. (C) WM/GM-FNs: K-means algorithm is used to create the WM and GM-FNs. (D) Atlas-FC: The average time series is extracted from a predefined atlas, and Pearson correlation is used to create the atlas-FC matrix. (E) Statistical Tests: A GLM model is fit to the FC elements and covariates provided by the user. After correcting for multiple comparisons, the elements in the FC matrix that are significant are identified.

The steps used by WhiFuN for checking data, preprocessing, creation of the WM/GM-FNs and corresponding FC, computing predefined atlas-based FC (atlas-FC), and finally, using statistical tests on the FC to find significant connections are described inFigure 1. InFigure 1A, the dataset is checked for missing images and MR parameters such as TR, voxel size, and number of time points. In case of any missing data or mismatched imaging parameters, the user is informed of these issues and is allowed to fix or discard corresponding data.Figure 1Bdemonstrates the sequence of preprocessing steps as performed by WhiFuN. Then, the creation of WM/GM-FNs is demonstrated inFigure 1C, and the computation of Atlas-FC is elucidated inFigure 1D. Finally,Figure 1Eshows how WhiFuN computes statistical testing with multiple comparison corrections on the FC to identify potential group differences or associations with behavioral measures.

### 2.2 Setting up WhiFuN

**Fig. 2.**
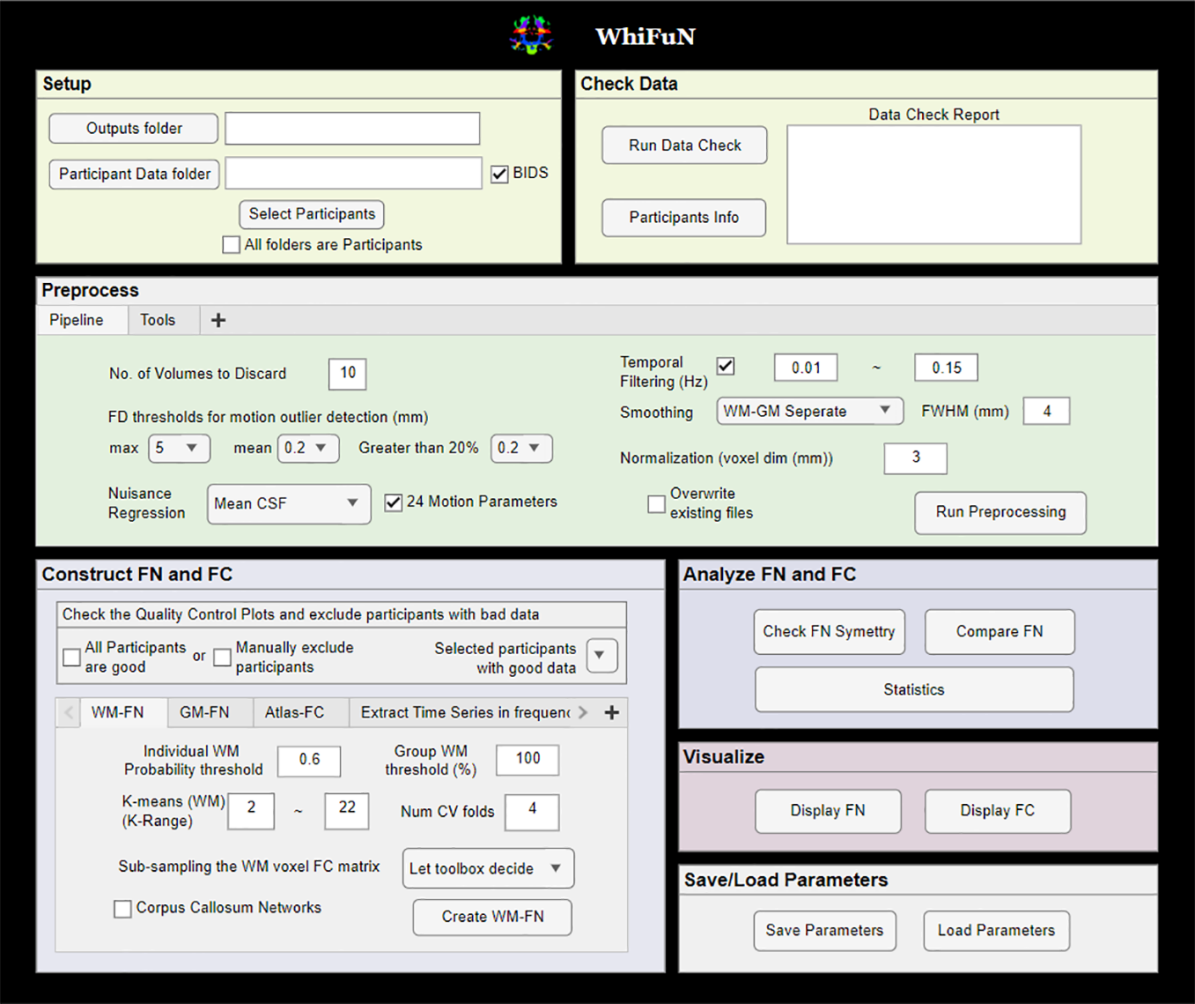
The GUI of the WhiFuN toolbox. The toolbox is divided into 6 subsections. Setup and check data: User provides the folder paths for functional and anatomical images and the output folder for the results, and WhiFuN checks the MRI parameters of the images and missing files. Preprocessing: The parameters associated with the preprocessing steps are defined here and preprocessing initiated. Functional networks (FNs) and Functional Connectivity (FC): WM/GM FNs are created, and Atlas-FC and frequency-specific time series are extracted here. Analyzing the FC and FNs: Group difference statistics of FC are computed. The WM/GM FNs generated can be compared to an atlas or predefined FNs using the*Compare FNs*module, and the degree of interhemispheric symmetry can be quantified using*check symmetry*module. Visualization: WM/GM FNs created can be visualized using the*Display FN*module, and the FC matrix can be visualized using the*Display FC*module. Save/Load Parameters: the paths and the parameters used during the preprocessing are saved as a*.mat file*which can be loaded back to avoid setting them again.

To run the toolbox, WhiFuN has to be downloaded into a folder, and the path of this folder added to MATLAB by either using ‘*setpath*’ under the ‘home’ tab in MATLAB (recommended as this needs to be done only once) or using theaddpathfunction (the user will have to do this each time MATLAB is started). Once the path information is added to MATLAB, typing ‘*whifun’*in the MATLAB command prompt will open the WhiFuN GUI window (Fig. 2).

As MATLAB functions ‘*niftiread*’ and ‘*niftiinfo*’ from the*Image processing toolbox*in MATLAB are faster than the ‘*spm_vol*’ function in SPM to read the nifti images, we have used the former functions in WhiFuN. Therefore, the*Image Processing toolbox*of MATLAB should be downloaded. For filtering the resting-state BOLD signals in time, the toolbox utilizes the Butterworth filter, implemented using the ‘*butter*’ function, which is included in the*Signal processing toolbox*. The ‘*regress*’ function is used to regress the nuisance signals. For performing the K-means clustering, the ‘*k-means*’ function and for the statistical testing ‘*regstats*’ is used, which are included in the*Statistics and Machine Learning Toolbox*. Finally, for the statistics module*mafdr*function is used for multiple comparison corrections which requires the*Bioinformatics toolbox*. These toolboxes must be downloaded and installed in MATLAB using the MATLAB add-ons feature.

### 2.3 Running data check

Before running the preprocessing and analysis steps, WhiFuN performs a data check. This module checks participants with missing anatomical or functional scans, consistency of voxel sizes across images, consistency of temporal resolution (TR), and the number of volumes (time points) of the functional scans. At the end of this step, a histogram and a bar plot corresponding to the above parameters across all participants are presented (seeSupplementary Figs. S1 and S2). The user must ensure that all participants in the dataset have the same parameters before proceeding to preprocessing. The check data module also generates and stores a*participant_list.csv*file with the above-mentioned parameters for all participants.

### 2.4 WhiFuN preprocessing

After the*Data check*is done and the user confirms that all file paths and image parameters are correct, the user can define the preprocessing parameters. This includes the number of fMRI volumes to discard (default 10), the thresholds on the maximum FD (default 5 mm) and mean FD (default 0.2 mm), and the threshold on which the FD values corresponding to more than 20% of volumes are greater (default 0.2 mm) to discard a participant due to excessive motion. Further, the user can choose from regressing the mean CSF BOLD signal (default), choosing the first few Principal components (default 5), or no CSF regression. There is a check box for regressing out the Friston’s 24 motion parameters. For spatial smoothing (here after referred to as smoothing), the user can choose to smooth the WM and GM regions separately (default), all voxels together with the FWHM specified (default 4 mm), or no smoothing. There is an option to include temporal filtering in the preprocessing with the specified filter range (default, 0.01 to 0.15) and, finally, the voxel size of the normalization output. There is an option to delete any preprocessed files created earlier by checking the ‘*overwrite existing files*’ checkbox or skipping the preprocessing steps if preprocessing was completed previously.

The preprocessing pipeline starts by ‘*gun zipping*’ any*.gz*file and discarding initial volumes (default is 10) from the functional images, allowing for the magnetization to stabilize to a steady state (Caballero-Gaudes & Reynolds, 2017). Each preprocessing step is described briefly in the following sections.

#### 2.4.1 Realignment and framewise displacement

The raw functional volumes are realigned to the first image using SPM ‘*Realign (estimate and reslice)*.’ The head motion associated with each volume is estimated by evaluating how much each volume is transformed to match the first volume.*SPM-Realign (estimate and reslice)*saves the translations of each volume in x, y, and z directions in millimeters and the rotations of each volume in pitch, roll, and yaw in radians in a text file (rp_〈functional image name〉.txt).

The framewise displacement (FD) is also calculated by adding the pairwise difference between consecutive translational parameters and converted rotational parameters. The converted rotational parameters are created by assuming the brain to be a sphere with a radius of 50 mm (Power et al., 2012). Outlier volumes are identified and corresponding scans are excluded if the maximum FD is greater than a threshold (default is 5 mm), if the overall mean FD is greater than a threshold (default 0.2 mm), or if more than 20% of volumes are greater than a threshold (default 0.2 mm) (Parkes et al., 2018). WhiFuN allows the user to set the above-mentioned thresholds based on how much motion can be tolerated for a particular cohort (seeSupplementary Fig. S3).

#### 2.4.2 Tissue segmentation

The anatomical T1 images corresponding to the functional images that pass FD criteria are then segmented into WM, GM, and cerebrospinal fluid (CSF) regions using “*SPM Segment*.” This step also identifies the areas that belong to the skull and background, and the skull is stripped from the anatomical images by applying the “*SPM ImCalc*.” Segmentation also provides the deformation field, which will be used during “*SPM Normalization*” to transform the image from the participant’s native space to the standard Montreal Neurological Institute (MNI) space (Mazziotta et al., 1995).

#### 2.4.3 Co-registration

The anatomical scans are co-registered to functional images using the ‘*SPM co-registration (estimate)*’ module. Co-registration is necessary as the WM, GM, and CSF regions identified from the anatomical scans are used to determine the corresponding regions in the functional image.

#### 2.4.4 Nuisance variable regression

After co-registration of the anatomical and rsfMRI images, the CSF voxels in the rsfMRI image are identified based on a threshold. The SPM segmentation module provides a tissue probability map that assigns a probability value to every voxel based on the likelihood that a voxel belongs to CSF for every participant. Voxels with a CSF probability above a threshold of 0.95 are included in the individual CSF mask. Once the individual CSF mask is constructed, the average CSF time series (default) or the first few principal components (default 5) of all CSF voxels are chosen for regression. In addition, Friston’s 24 motion parameters are also regressed from the functional time series to eliminate motion-related noise (Friston et al., 1996;Yan et al., 2013). Friston’s 24 parameters are the three translational and three rotational parameters, the squares of them, the derivatives of them, and the squares of the derivatives. Regression is performed to remove the nuisance signals from the functional time series.

#### 2.4.5 Temporal filtering

After regression, the voxel time series from every voxel are filtered using a Butterworth bandpass filter of second order in the range specified by the user (default 0.01 to 0.15 Hz). A default upper cutoff of 0.15 Hz was used as the peak amplitude of signals from WM was found to be close to 0.10 Hz for some of the WM-FN, suggesting that WM functional signals may exhibit meaningful fluctuations in higher frequency ranges (Peer et al., 2017).

#### 2.4.6 Smoothing

After temporal filtering, the images are smoothed using a Gaussian filter of full-width half maximum (FWHM) specified by the user (default = 4 mm) using the “*SPM smooth*” module. The user can select one of the following smoothing options: 1) Smooth GM and WM separately (default) to ensure that the WM and GM signals do not mix (Peer et al., 2017). 2) The regular SPM smooth that smooths all the voxels in the brain together and 3) no smoothing.

#### 2.4.7 Normalization to MNI space

Normalization is performed after smoothing, where images are transformed from the participant’s native space to the standard MNI space of voxel size specified by the user (default 3 mm) using the ‘*SPM Normalize (write)*’ module. The deformation field generated during segmentation is used for normalization. The normalization step has a bounding box parameter that determines the field of view of the image with respect to the anterior commissure. The default bounding box parameter set in SPM is slightly expanded to [-90 -126 -72; 90 90 108] to ensure that all brain tissue regions are included.

### 2.5 Post preprocessing steps

After the preprocessing is done, there is a tools tab using which the user can further observe or change the preprocessing files created. Using the*Determine Framewise Displacement Thresholds,*the user can decide whether the current FD thresholds are acceptable or change them by looking at how many participants are rejected based on the different FD thresholds. If co-registration fails for some participants, for these participants, the user can manually co-register the anatomical and functional images using ‘*manually coregister participants*’ button and finally if a certain preprocessing step is not done as desired for a participant, the user can delete the preprocessing files for that participant from any intermediate step using the ‘*Delete preprocessing from a particular step*’ option and again rerun the preprocessing.

### 2.6 Checking quality control plots

After the preprocessing steps are completed, quality control (QC) plots (Di & Biswal, 2023) are saved in the ‘Quality Control’ folder. It is recommended that the user checks the QC plots and ensures that the preprocessing steps are successfully completed. The quality control plots are shown inFigure 3and briefly described below.

**Fig. 3.**
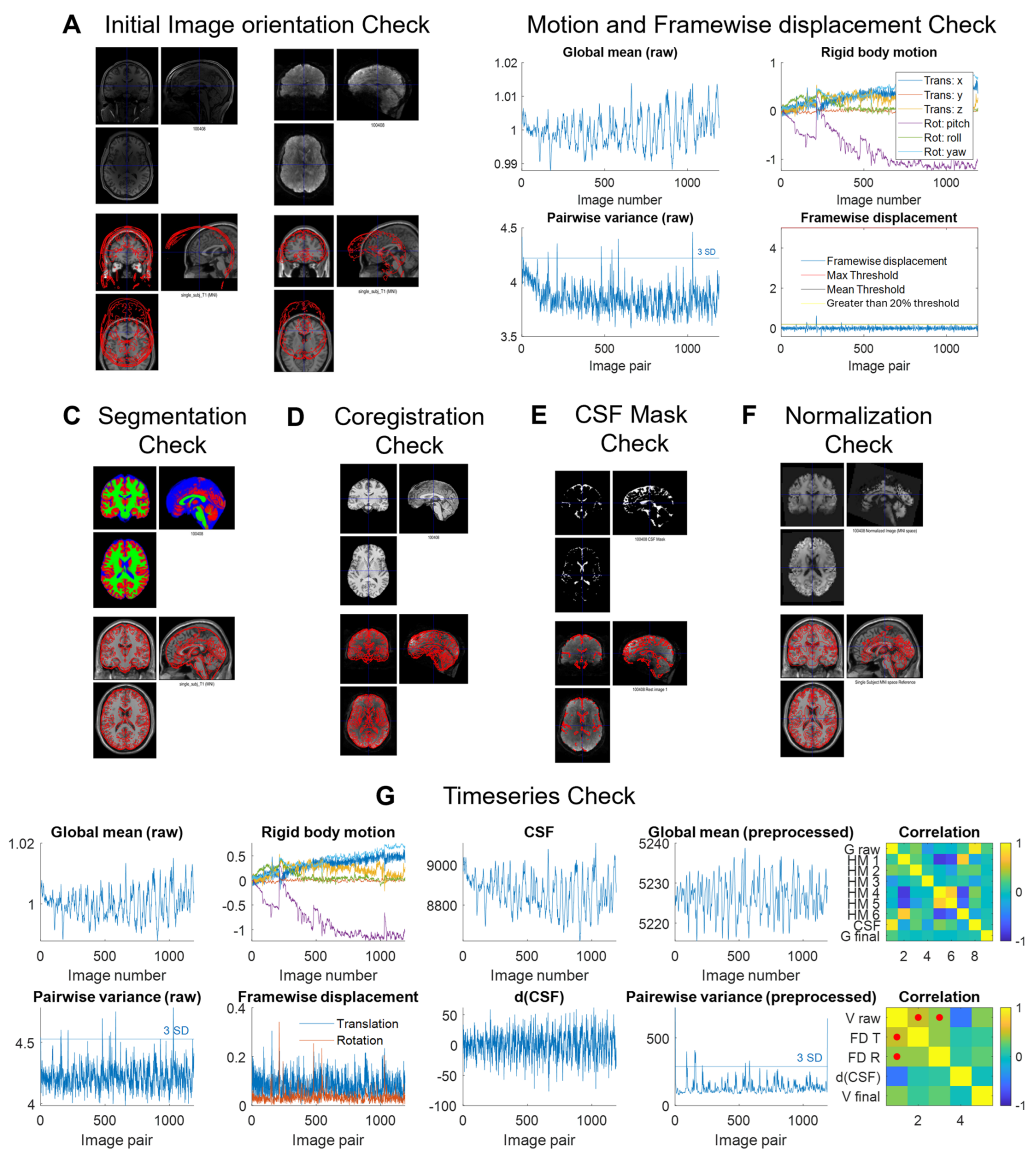
Quality control (QC) plots for a representative participant. (A) QC for the initial orientation of the raw images to the MNI reference template. (B) Global mean, 6 motion parameters, pairwise variance between consecutive volumes, and the FD plotted across time points. (C) Segmentation QC, Red = GM, Green = WM, and Blue = CSF. (D) QC for checking the co-registration between the anatomical and functional images. (E) CSF Mask alignment with functional image check (F) The normalized image with its contour plotted on the reference MNI template. (G) Timeseries plot showing the global time series before preprocessing, the 6 motion parameters, the mean CSF time series, the global mean signal after preprocessing, and a correlation matrix correlating these signals. The same is repeated for pairwise variance across consecutive scans instead of the global mean in the 2^nd^row. Figures generated using the WhiFuN toolbox.

#### 2.6.1 Initial orientation of the anatomical and functional scans before preprocessing

The raw anatomical and functional images are stored as*png*files in the quality control folder with the image contours on the standard MNI reference template. The user must check the initial position and orientation of the images with that of the MNI space. If the anatomical image is far from the MNI template or orientated differently, then the user must reorient the image manually to the template direction and reset the origin to the anterior commissure (Di & Biswal, 2023).

#### 2.6.2 Motion parameters and FD values after realignment

This plot shows the global mean of the raw unprocessed functional, the 6 motion parameters computed during realignment, the pairwise variance computed for the above-mentioned global mean signal, and the FD computed along with the thresholds. The user can observe from these plots why WhiFuN rejected certain participants. By default, WhiFuN rejects participants with excessive motion, and the user does not have to take any additional steps.

#### 2.6.3 GM, WM, and CSF tissue segmentation results

WhiFuN saves the segmentation outputs with the GM, WM, and CSF probabilities overlapped on the same image in red, green, and blue colors respectively. It also draws the contour of the segmentation output on the reference MNI space to check if the alignment of the normalized anatomical image with respect to the MNI template is correct. Users must observe the images for each participant and note any misclassification of the tissues. If any of the tissues are misclassified, then that participant may be discarded. Also, if brain lesions or image quality issues are noticed in the anatomical image, this participant’s data should be discarded.

#### 2.6.4 Contours of the anatomical image over the functional image after co-registration

Here, the anatomical image is shown with the functional image, and the contour of the anatomical image is overlaid on the functional image in red. The user must check the orientation and alignment of the anatomical and the functional images. If the images are not aligned, the user can manually align them using the ‘*manually coregister participants*’ module.

#### 2.6.5 CSF mask based on which the average CSF time series is computed

Here, the CSF mask extracted using a threshold of 0.95 on the tissue probability map of CSF is plotted with the functional image along with the contour of the CSF mask on the functional image. The user must check the alignment of the CSF mask and the functional image. It is observed in some cases that no voxels satisfy the 0.95 threshold. In such cases, the anatomical image must be rechecked, and the user may discard this participant.

#### 2.6.6 Effects of regression and temporal filtering

The global (average) signal before and after regression of the motion parameters and the CSF signals is stored. The user may observe that any upward or downward trends and artifacts due to motion are removed after regression.

The global signal before and after temporal filtering and the filter’s magnitude and phase in the frequency domain are also stored. Here, the user may observe how very high or low-frequency signals are removed during filtering. These plots are just for observing the effects of regression and temporal filtering on the global signals. The user does not need to take any additional steps here.

#### 2.6.7 Effects of smoothing GM and WM separately

One may observe that when the GM and WM voxels are smoothed separately, the cortex gets a number of small holes as these voxels were neither identified as GM nor WM. If the user observes a large number of holes in the cortex, the participant may have to be discarded. These are generated as there is CSF in the sulci. If there is a big difference between the voxel size of the anatomical and functional image, then a lot of these holes might get generated. This QC plot notifies the user about the holes.

#### 2.6.8 The contour of the normalized image overlaid on the reference MNI template

The normalized functional image is plotted with the reference MNI template image with the contour of the normalized functional on the reference MNI template. The user must ensure that the spatial alignment of the normalized functional image with that of the MNI template is correct.

#### 2.6.9 Time series check

The user can observe the associations between the global and noisy signals at this step. WhiFuN plots the raw unprocessed global signal, the six rigid motion parameters computed during realignment, the CSF signals, the global mean of the preprocessed image, and the correlation of all the above-mentioned signals. WhiFuN also plots pairwise variance between consecutive volumes (similar to DVARS;Power et al., 2014) for the unprocessed image, the framewise displacement, the derivative of CSF signals, the pairwise variance between consecutive volumes for the preprocessed image, and the correlation of these signals. The user must check the correlation plots and observe if the preprocessed global signal is correlated to the noisy signals. If the correlation of the global signal corresponding to the final preprocessed signal with any of the noisy signals is significant, the user may exclude the participant.

It is recommended that the user examines all QC plots of all the preprocessing steps to remove outlier participants from further analysis.

### 2.7 Construct functional networks and functional connectivity

After excluding the outlier participants and selecting the final list of participants to analyze further, the WM and GM-FNs can be constructed using the FN and FC module. Group masks are created first to identify the WM or GM voxels across the participants. The K-means algorithm clusters the voxels in WM and GM to create the WM and GM-FNs based on voxel-based FC. Once the FNs are constructed, the averaged time series corresponding to every FN is extracted and stored as*.mat*files.

WhiFuN requires that the user specifies theWM groupthreshold %(default 100%),Individual  WM probabilitythreshold(default 0.6), and K-value range (default 2 to 22) to construct the FNs. For every K-value in the range, the average dice coefficient and the distortion value are computed to help the user find the optimal K-value. An optimal K-value has a high average dice coefficient and low distortion value. Once the user enters the optimal K-value, WhiFuN computes the FNs and extracts the average time series corresponding to each FN for every participant.

WhiFuN stores the computed FNs as*nifti*files with the name*WM_clustering_K <*value of K used*>.nii*for WM-FNs and*GM_clustering_K<*value of K used*>.nii*for GM-FNs. The average BOLD time series of every WM and GM network for every participant are stored as a*network_avgts_wm_K<*value of K used*>.mat*and*network_avgts_gm_K<*value of K used*>.mat*file in the specified output folder.

The following sections describe the pipeline and algorithm used to construct the WM and GM FNs.

#### 2.7.1 WM and GM group mask construction

A stringent threshold is used to create the WM group mask to ensure that no GM voxels influence the WM signal. All voxels for which at leastWM group threshold %(default 100%) of participants have WM probability greater than aIndividual  WM probability threshold(default 0.6) are included in the WM group mask. We picked 0.6 as the default value forIndividual  WM probability thresholdto ensure that only highly probable WM voxels are included in the mask. If a voxel has a WM tissue probability of 0.6 or more, there is very less chance of it being GM or CSF. For the GM, the threshold is kept less stringent; all voxels for which at leastGM group threshold% (default 20) of participants classified as GM and not included in the WM group mask, are included in the GM group mask. SPM can classify specific GM subcortical structures as WM due to their high iron content (Lorio et al., 2016). Thus, sub-cortical regions identified by the Harvard-Oxford Atlas (Desikan et al., 2006) are included in the GM mask even if SPM identified them as WM. Moreover, voxels that have no values in more than 20% of participants are also removed from the group masks (Peer et al., 2017). The Group WM and GM masks constructed here are only used to compute the FNs using the K-means algorithm described inSection 2.7.3.

#### 2.7.2 Functional connectivity

The clustering of the voxels in WM/GM group masks is done based on the voxel level FC. Thus, FC matrices are created for both WM and GM separately, considering the voxels identified in WM and GM group masks, respectively.

The FC matrix is created by correlating every voxel with every other voxel in the group mask. This results in an FC matrix of sizeN×NwhereNis the number of voxels in the group masks. This FC matrix will be given to the K-means algorithm for constructing the FNs. Any given voxel in the group mask will be assigned to one of the FN based on its complete FC (correlation between the given voxel and every other voxel in the group mask). Depending on the group mask thresholds and the resolution of the preprocessed functional images, the value ofNcan be between 5,000 to 20,000 for WM and 50,000 to 70,000 for GM. SinceNcan be a huge number matrix of size,N×Ncan use up a lot of the computer’s memory. To mitigate this issue, instead of using the complete FC, a subsampled FC can be used for every voxel. Where for every voxel in the WM, its correlation with every alternate voxel will be considered, and for the GM, its correlation with every 3^rd^voxel will be considered. Thus, the final matrix given to K-means is of size$N\times N_{s}$where$N_{s}$is the number of subsampled voxels. Based on the user’s computer memory to compute the FNs, it is possible that one may not need to perform subsampling (If no subsampling is performed$N_{s}\;=N$). WhiFuN will determine whether subsampling is necessary or not based on the available memory.

#### 2.7.3 Functional networks

The WhiFuN toolbox can create both WM and GM-FNs. Based on the user’s choice, the respective FC matrix (GM or WM) is fed into the K-Means algorithm to identify FNs. The rows of the matrix are considered data points, and the columns of the matrix are considered features. For instance, K-Means clustering takes place with$N_{WM}$data points in an$N_{s_{WM}}$dimension space for the WM-FNs ($N_{wm}$and$N_{s_{WM}}$being the number of voxels in WM group mask and subsampled voxels, respectively) and$N_{GM}$data points in a$N_{s_{GM}}$dimension space for the GM-FNs. The K-Means algorithm clusters the data points intoK(specified by the user) clusters. At the end of K-Means clustering, every voxel in the mask is assigned to one of theKclusters. As K-Means is applied to the FC matrix, Pearson correlation is used as the distance measure used to compute the clusters. Refer toSupplementary Figure S5for a graphical representation of finding the optimal K-value and constructing the FNs.

A cross-validation approach is applied to determine the optimal number of clusters (K). The FC matrix is divided into$n_{cv}$different sub-folds (default 4) by selecting a subset of features. Each sub-fold matrix is of size$N_{WM}\;\times \;\;\left(\frac{N_{s_{WM}}}{n_{cv}}\right)$for WM and$N_{GM}\;\times \;\;\;\left(\frac{N_{s_{GM}}}{n_{cv}}\right)$for GM. The K-means algorithm computes theKclusters for each sub-fold independently. Ideally, if theKvalue is equal to the actual number of FNs in the data, the clusters computed would be the same irrespective of the subset of features chosen. Thus, to determine the optimal value ofK, for every value ofK, the clusters formed with the different sub-folds are compared to each other.

As the clustering algorithm randomly assigns labels to the clusters, the assigned labels cannot be directly used to determine the similarity between two sets of clusters. To solve this problem, an adjacency matrix ($Adj_{WM}$for WM and$Adj_{GM}$for GM) is created for each cluster label that represents whether or not two voxels belong to the same cluster.

$\begin{array}{l} Adj_{WM}(i,j)=\text{​}\;1,\;\text{if }i\;\text{and }j\;\text{belong to the same cluster},i=1,2,...,\;\\ N_{WM},\;j=1,2,...,N_{WM} \end{array}$

$Adj_{WM}(i,j)=\text{​}\;0,\text{ if }i\text{ and }j\;\text{belong to a different cluster}\;$

The$Adj_{WM}$is a large matrix of size$N_{WM}\;\times N_{WM}$. (A similar adjacency matrix can be created for GM). This large matrix can take up a lot of memory, and to help facilitate computing this matrix, small chunks of 100 voxels are processed at a time to create a smaller100 ×100adjacency matrix. IfKequals the actual number of FNs in the data, then ideally, the adjacency matrix across all the different folds should be the same for a particular chunk of 100 voxels. The similarity between adjacency matrices for every pair of folds is calculated using Dice coefficients. Thus, a Dice coefficient is obtained for every pair of sub-folds and every chunk of 100 voxels. All these Dice coefficients are averaged, giving one averaged Dice coefficient for a particular value ofK. To determine the optimal value ofK,a grid search method is used. A range ofKvalues is specified by the user (the default range is from 2 to 22), and the average dice coefficient for every value ofKis computed. A plot of the average dice coefficients versusKallows the identification of stable solutions as local peaks on the plot.

The ‘distortion’ measure that calculates on average how far the data points are from their respective cluster center is also computed for every value ofK. The lower the distortion value, the more optimal value ofK. So, an optimal value ofKwould be that which has a lower distortion value and a higher average dice coefficient value. Based on these two measures, the user can choose the optimalK.

Once theKis chosen by the user, WhiFuN will create the clusters using the K-means algorithm with distance metric ‘correlation’ and repetition of 10 (which means the K-means algorithm will run 10 times with different initial conditions, and the solution with the smallest distortion value will be saved). These clusters of brain voxels constructed by the K-means algorithm are the FNs.

#### 2.7.4 Corpus callosum FNs

Corpus callosum (CC) is the largest WM bundle connecting the human brain’s two hemispheres, containing more than 250 million axons (Nolte, 2002). It has been shown that the CC plays a crucial role in transmitting sensory-motor and cognitive information between two cerebral hemispheres and that it is functionally and structurally connected to WM-FNs (P. Wang et al., 2021). Thus, we included the method proposed byP. Wang et al. (2021)that computes the CC-FNs on brain regions labeled as corpus callosum by the Mori atlas (Mori et al., 2008).

Once the WM-FNs are created, WhiFuN can be used to find the CC regions that are maximally correlated with the average time series extracted from the WM-FNs. To achieve this, CC-voxels labeled from a predefined atlas (Mori et al., 2008) are removed from the WM-FNs. Then, the time series from every CC voxel is partially correlated to every WM-FN while controlling for the effect of the average time series of otherK−1WM-FNs. This gives a partial correlation coefficient between every CC-voxel andKWM-FNs for every participant. The correlation scores are then Fisher-z transformed. For a particular CC voxel, Fisher-z transformed correlations significantly different from 0 across participants are found using a one-sample t-test, resulting in a statistical t value for each WM-FN. To assign the CC-voxels to one of the WM-FNs, the WM-FN with the maximum t-statistic for a particular CC-voxel is assigned to that CC-voxel. Thus, the CC FNs created use a winner take all strategy, where each voxel in the CC is assigned to only one WM-FN. This removes the possibility of two different WM-FNs interacting with a certain sub-region of the CC. However, this can be mitigated by using the FC to compute the average BOLD time series corresponding to every WM-FN and correlate it with the average BOLD time series extracted from every subregion of the CC.

Under the WM tab, there is an option to compute the CC FNs associated with the WM-FNs. If this option is chosen, the CC region is excluded from the WM group mask using the CC atlas (Mori et al., 2008). Then, the CC is divided into sub-regions based on the connectivity of the WM-FNs with the CC sub-regions. These CC sub-regions are stored as a*nifti*file named*cc_networks_from_WM_K<*value of K used for the WM-FN*>.nii*, and the averaged time series is extracted from each CC sub-region is stored as*cc_avg_ts_<*value of K used for the WM-FN*>.mat*.

### 2.8 Atlas functional connectivity

The user can also extract the average time series from predefined atlas ROIs. A few JHU atlases (Mori et al., 2008;Oishi et al., 2009) are included in WhiFuN for ROI-based analysis. There is also an option to manually select any other atlas (only nifti images or nifti gzip files are currently accepted). Once the atlas is defined, WhiFuN extracts the voxels corresponding to every ROI and computes the average time series corresponding to every ROI. The average time series for every ROI, for every participant, is stored as <atlas_name>_*reg_ts*.*mat*file.

Recent papers have indicated differences between patient and healthy controls in FC values computed from time series filtered in specific frequency bands (Jiang et al., 2019;X. Li et al., 2022). WhiFuN can also extract the time series filtered with specific frequency bands specified by the user. WhiFuN saves the averaged time series filtered with specific frequency bands as <atlas_name>_<frequency_band>*_avgts.mat.*

### 2.9 Creating the FC matrix

The average time series files stored after the creation of WM, GM, CC FNs, and/or atlas time series are used to compute the FC matrix. Using Pearson correlation between every pair of average time series an  ×  nmatrix (wherenis the number of WM/GM/CC FNs or the number of ROIs in the predefined atlas) can be computed for every participant. This FC can be used to compute the graph’s theoretical measures to explore differences between cohorts using the Brain Connectivity toolbox (Rubinov et al., 2009).

### 2.10 Analyzing the FNs and FCs

This module analyzes the FNs and FC created during the*Construct FNs and FC*module by finding significant connections using statistical tests in FCs, checking how symmetric the FNs are across the two hemispheres, or comparing the FNs computed by WhiFuN with any other predefined atlas to understand the network labels.

#### 2.10.1 Compare FNs

The ‘*compare FNs*’ module can be used to compare two sets of FNs. This function compares the FNs based on the Dice coefficient for two sets user specified FNs. WhiFuN plots an$n_{1}\times n_{2}$matrix consisting of the Dice coefficient values calculated between every FN from the first set with that of the second set, where$n_{1}$is the number of FNs in the first set and$n_{2}$is the number of FNs in the second set. This feature is helpful if the user wants to label the FNs by comparing them to a predefined atlas.

#### 2.10.2 Check FN symmetry

Using the ‘*Check FN*Symmetry’ module, WhiFuN can calculate how symmetric the FNs are between the two hemispheres. The user can compute the symmetricity for all the WM-FNs simultaneously or separately for every WM-FN. This function first divides the given FN into the left and right hemispheres.

To calculate the total symmetricity value for all the FNs at once, the method proposed byPeer et al. (2017)is used. Briefly, every voxel in the left atlas that does not have a corresponding symmetric right counterpart and vice versa is discarded. For the voxels that are not discarded, an adjacency matrix is created for each hemisphere separately. The Dice coefficient between the left and the right hemisphere adjacency matrix is reported as symmetricity score for all the FNs.

The symmetricity could be calculated separately for every FN as well. First, the Dice coefficients between every FN on the left versus every FN on the right are computed. There can be instances where an FN is not symmetric to itself but is present on only one hemisphere and is symmetric to another FN (e.g., inFig. 7, WM1 is symmetric to WM6 while WM2 is symmetric to itself). The*check FN symmetry*module outputs a plot showing the Dice coefficients computed by the FNs in the left hemisphere versus those in the right hemisphere. The higher the Dice coefficients, the more symmetric the FNs are.

#### 2.10.3 Statistics

WhiFuN can perform statistical tests on WM, WM-GM, and WM-CC FC (using MATLAB’s*regstats*function) to find significant differences between groups of participants or how well a particular FC can explain a behavioral score. To avoid false positives due to multiple comparisons, false rate discovery (FDR) is computed using ‘*mafdr*’ (the Benjamin and Hochberg method) or Bonferroni correction. Corrections for multiple comparisons are based on the number of FC connections between every FN pair. The False discovery rate (FDR) correction is done by considering thep-values obtained from the regstats function for every FC element. The FDR is computed by giving the$n_{total}$different p-values obtained from every FC element using the MATLAB function*mafdr*. For WM-FC, if there are$n_{WM}$WM-FN,$n_{total}=\frac{n_{wm}\times (n_{wm}-1)}{2}$. A similar approach is adopted for GM and CC FDR corrections. For Bonferroni correction, the corrected alpha ($\alpha_{corrected}=\frac{\alpha }{n_{total}}$) is computed by dividing the alpha value by the number of tests that are performed.

**Fig. 4.**
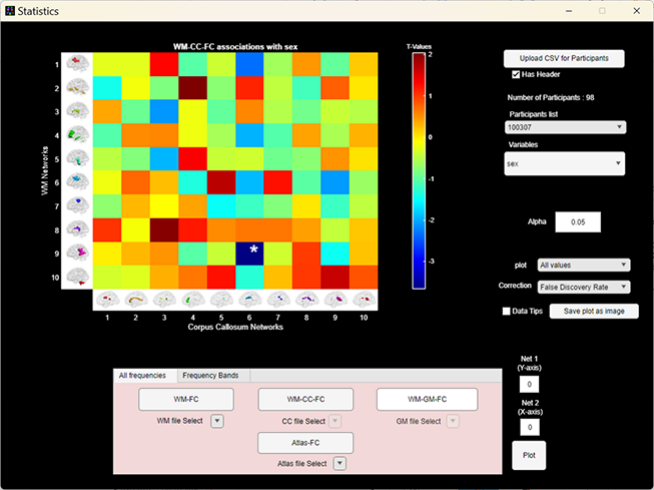
GUI of the*Statistics*module. After computing WM/GM/CC FNs, the user can upload a CSV file consisting of the covariates for every participant and perform statistical tests to identify group differences or associations with behavioral measures. The color bar represents the t-values corresponding to WM-CC FC sex differences.

Figure 4shows the WhiFuN statistics module. WhiFuN lets the user upload a CSV file containing the participant name/identifier in column 1 and other predictors (participant group, demographics, behavioral scores, participant head motion parameters, etc.) in the other columns. WhiFuN plots the t-values corresponding to every connection in the FC and highlights the statistically significant connection. The statistical significance ‘*alpha*’ value can be specified (default 0.05).

### 2.11 Visualizing the FN and FC

This module can be used to visualize the WM, GM or CC FNs, and the FC matrix.

#### 2.11.1 Display FN

After the WM or GM-FNs are constructed, a visual display of the FNs can be done by using the*display FN*function (seeFigs. 7and8).

#### 2.11.2 Display FC

The user can visualize the FC matrix by using the Display FC button in the Visualization module. This allows the user to visualize the mean and the standard deviation FC matrix across participants within WM-FNs, between WM and GM-FNs, and between WM and CC FNs in addition to the participant-level FC matrix for each participant using the drop-down menu (seeFig. 5). By dividing the participants based on groups (by manually assigning participants to groups or uploading a CSV file), the user can also view the mean/std FC corresponding to the participants of a group. While the t-test module elucidates the significantly different connections between two groups (in this case, males and females), the Display FC module can be used to observe the FC values.

**Fig. 5.**
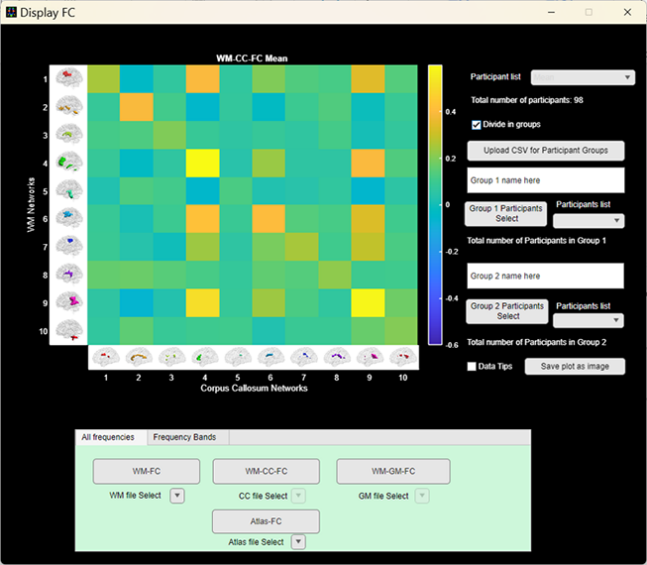
GUI for the*Display FC*module. Currently displaying the Mean WM-CC-FC computed across all participants. The user can see the mean, standard deviation, or individual participant FC or Atlas-FC using this module. The color bar represents the Pearson correlation coefficients.

## 3 Results: Application of WhiFuN to Human Connectome Data

### 3.1 Dataset and processing

To demonstrate the application and usage of WhiFuN, we use the publicly available Human Connectome Project (HCP) dataset (Van Essen et al., 2012,^2013^) of 100 unrelated participants (46 males and 54 females, mean age29.1  ±  3.7years). The functional data corresponding to rest1 (LR) scans were used to create the WM and GM-FNs, and the rest2 (LR) scan/session was used to evaluate reproducibility. The unprocessed raw images were preprocessed using WhiFuN with the default parameters for the preprocessing steps. Two participants were excluded due to high levels of head motion: one participant was discarded as it had maximum FD greater than 5 mm, and the other was discarded as it had an FD greater than 0.2 mm for more than 20% of volumes.

### 3.2 Constructing of FNs

**Fig. 6.**
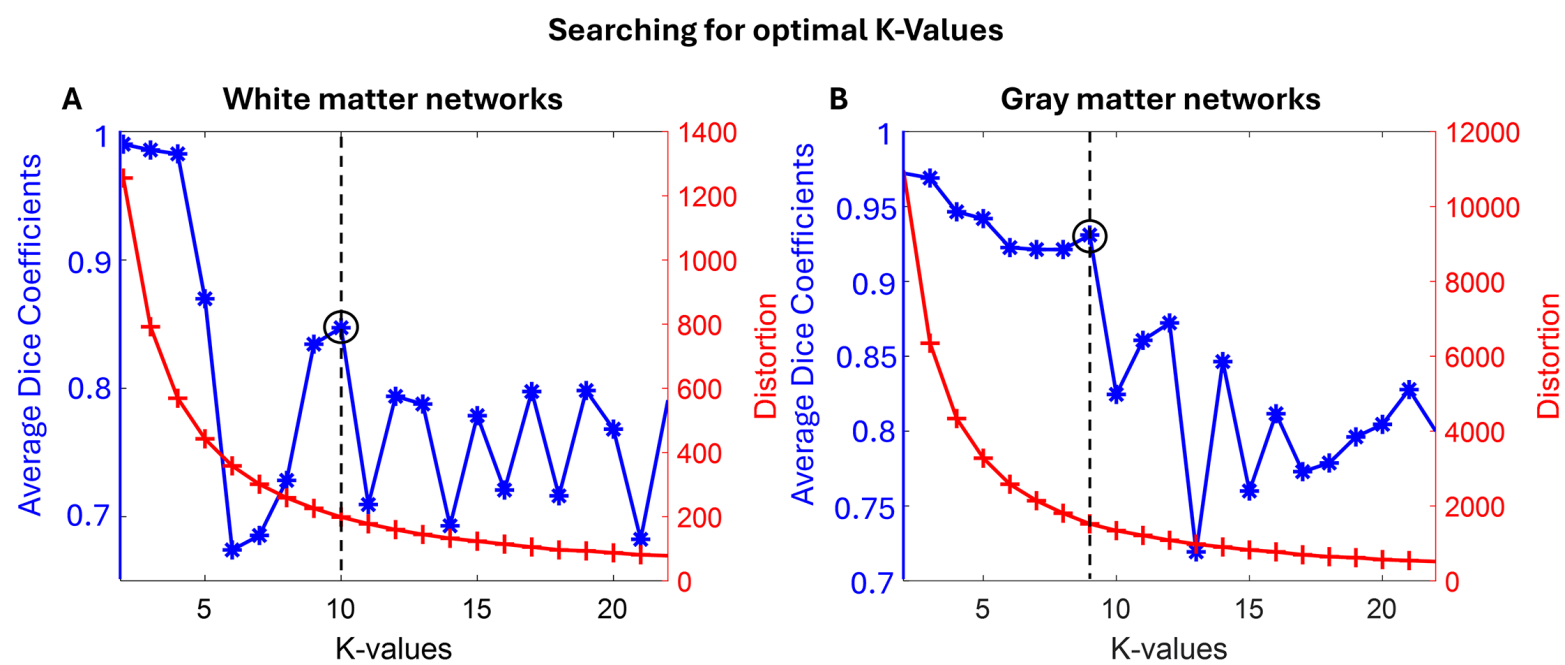
Finding the optimal number of FNs (K value). (A) for WM-FNs and (B) for GM-FNs. K = 10 and 9 were chosen for WM-FNs and GM-FNs respectively.

WM-FNs were created by identifying voxels in WM for which 100% of participants had WM probability greater than 0.6. A total of5,319WM voxels were present in the WM mask. On a computer with 64 GB of RAM, we did not have to subsample the WM voxels, and a correlation matrix of5,319×5,319was given as input to the K-means algorithm. An optimalKvalue was searched between 2 and 22. Based on the average Dice coefficient and elbow plot (Fig. 6A), aKvalue of 10 was chosen as the optimalKand 10 WM-FNs were constructed. The CC-FNs were also created, and the FC between the WM-FNs and the CC sub-regions was computed. A total of51,617voxels were detected in the GM, and a subsampling strategy was used for GM, and a correlation matrix of51,617×5,714was given as input to the K-means algorithm. After searching for the optimal K value for the GM-FNs between 2 and 22, an optimal value of K = 9 was chosen for the GM-FNs (Fig. 6B).

The WM-FNs constructed by WhiFuN are presented inFigure 7A. The*compare FN*feature of WhiFuN was used to compare the WM-FNs with the JHU-DTI-81 atlas (file name JHU ICBM labels 1 mm) (Mori et al., 2008) (https://identifiers.org/neurovault.collection:264) to name the WM-FNs. Moreover, WhiFuN was also used to compare the CC-FNs with the seven sub-regions of CC (Witelson, 1989). To further refine the analysis, we divided each of these seven sub-regions into left and right hemispheric components, resulting in a total of 14 sub-regions.Table 1shows the names of the JHU-regions of the WM-FNs constructed by WhiFuN and the corresponding CC-FNs. SeeSupplementary Figure S7for the Dice coefficients computed between WM-FN and JHU-DTI-81.

**Fig. 7.**
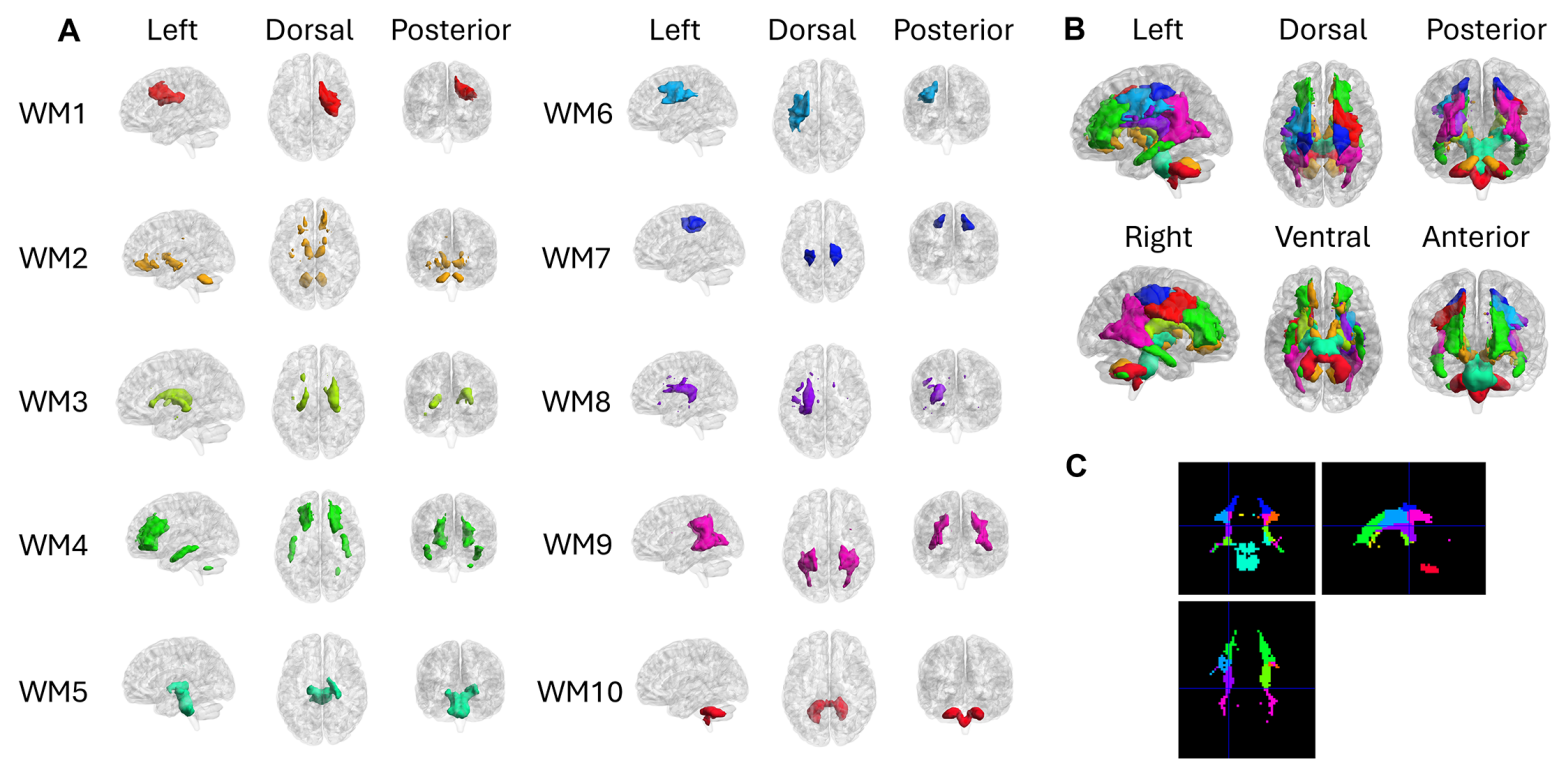
(A) WM-FNs constructed by WhiFuN for the HCP-100 dataset. Anatomical labels of overlapping JHU atlas regions are provided inTable 1. (B) Composite view of the WM-FNs on glass brain. (C) Composite WM-FNs in orthogonal slice views.

**Table 1.** Regions corresponding to WM-FNs and CC-FNs constructed.

WM-FNs	JHU-DTI-81 atlas overlapping with WM-FNs	CC-FNs	Corresponding Corpus callosum Regions
WM1	Right Superior Corona Radiata, Right Superior longitudinal fasciculus	CC1	Right Anterior midbody
WM2	WM regions in the cerebellum, a part of the left and Right cerebellar peduncle, a part of the left and right posterior and anterior limb of the internal capsule, and parts of forceps minor	CC2	Right Splenium, Right genu, Right Rostral body
WM3	Right anterior, posterior limb, and retro lenticular part of the internal capsule, a part of the Left posterior limb of IC, and Right external capsule	CC3	Left Rostrum
WM4	Right and left anterior corona radiata	CC4	Left and Right genu, Left Rostral body,
WM5	Pontine crossing tract, Right and left Cortico-spinal tract, and right and left cerebellar peduncle	CC5	Right Isthmus
WM6	Left Superior Corona Radiata and left Superior longitudinal fasciculus	CC6	Left Anterior, posterior and midbody, left Rostral body
WM7	A different part of Superior Corona Radiata (as compared to WM1)	CC7	Right Isthmus, Right Posterior midbody
WM8	Left anterior, posterior limb, and retro lenticular part of the internal capsule, and Right external capsule	CC8	Left isthmus
WM9	Left and right posterior corona radiata and left and right posterior thalamic region	CC9	Left and Right Splenium
WM10	middle cerebellar peduncle	CC10	A different part of Right Anterior midbody (as compared to CC1), and Left Splenium (as compared to CC9)

**Fig. 8.**
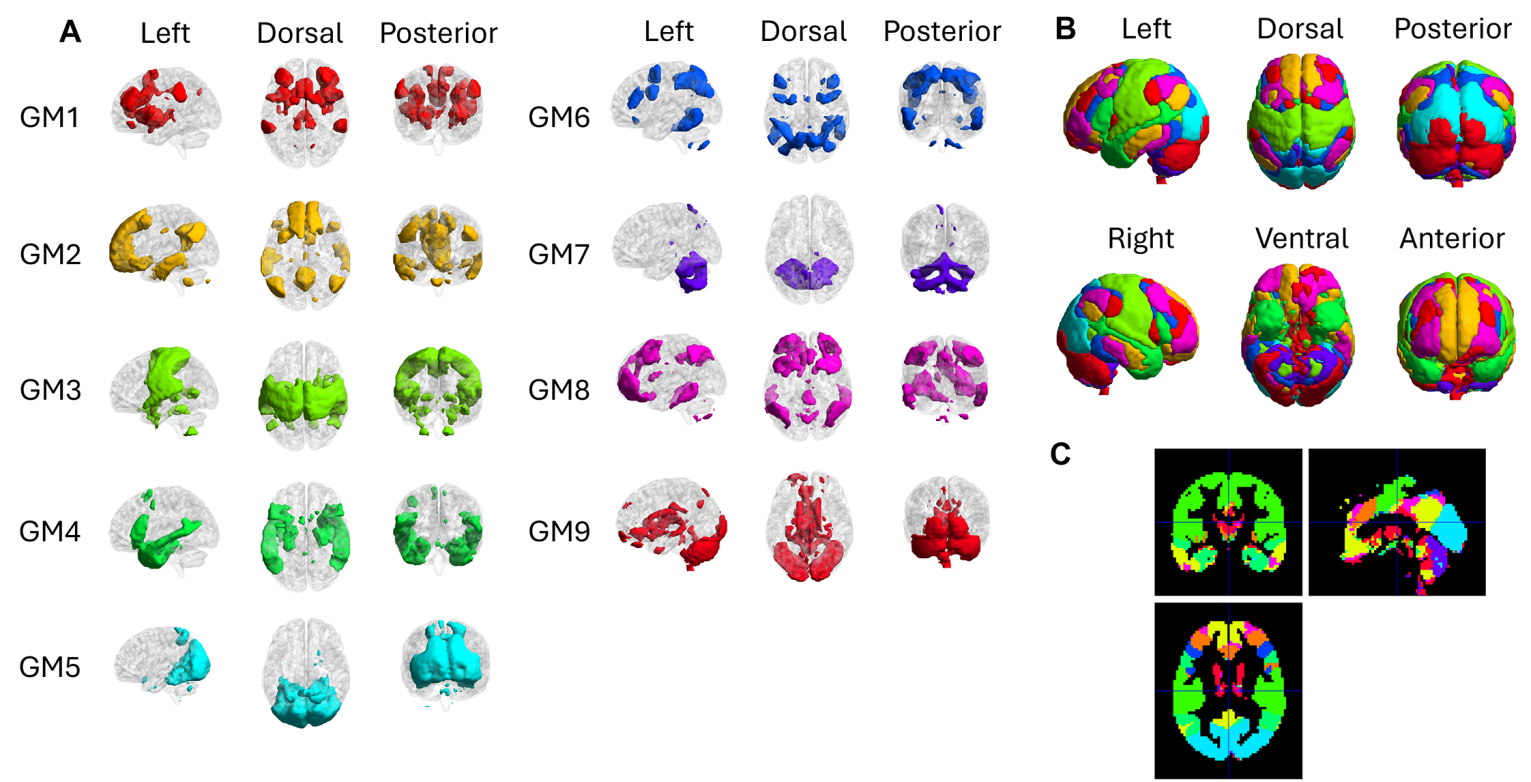
(A) GM-FNs constructed by WhiFuN for the HCP-100 dataset. (B) Composite view of the GM-FNs on glass brain. (C) Composite GM-FNs in orthogonal slice views.

Figure 8Ashows the GM-FNs constructed by WhiFuN. Using the*compare FN*feature of WhiFuN, the GM-FNs identified by the toolbox were labeled according to the Yeo 7 resting-state networks (Yeo et al., 2011); seeTable 2. Note that the Yeo networks do not contain the cerebellum or the subcortical regions of the brain, and GM4 and GM7 were named by manually inspecting the brain regions. SeeSupplementary Figure S6for the dice coefficients found by comparing the GM-FNs constructed by WhiFuN with the Yeo atlas.

**Table 2.** Yeo networks corresponding to the GM-FNs.

GM-FNs	Yeo atlas regions overlapping with GM-FNs
GM1	Ventral attention Network
GM2	Default mode network (DMN)
GM3	Somato Motor Network
GM4	Limbic-DMN Network
GM5	Visual Network
GM6	Dorsal attention network
GM7	Cerebellum – subcortical Network
GM8	Fronto-Parietal Network
GM9	Cerebellum Network

The symmetricity of the GM and WM FNs was quantified using the*check FN symmetry*module, and the output of the module is shown inSupplementary Figure S8.

### 3.3 Sex differences

**Fig. 9.**
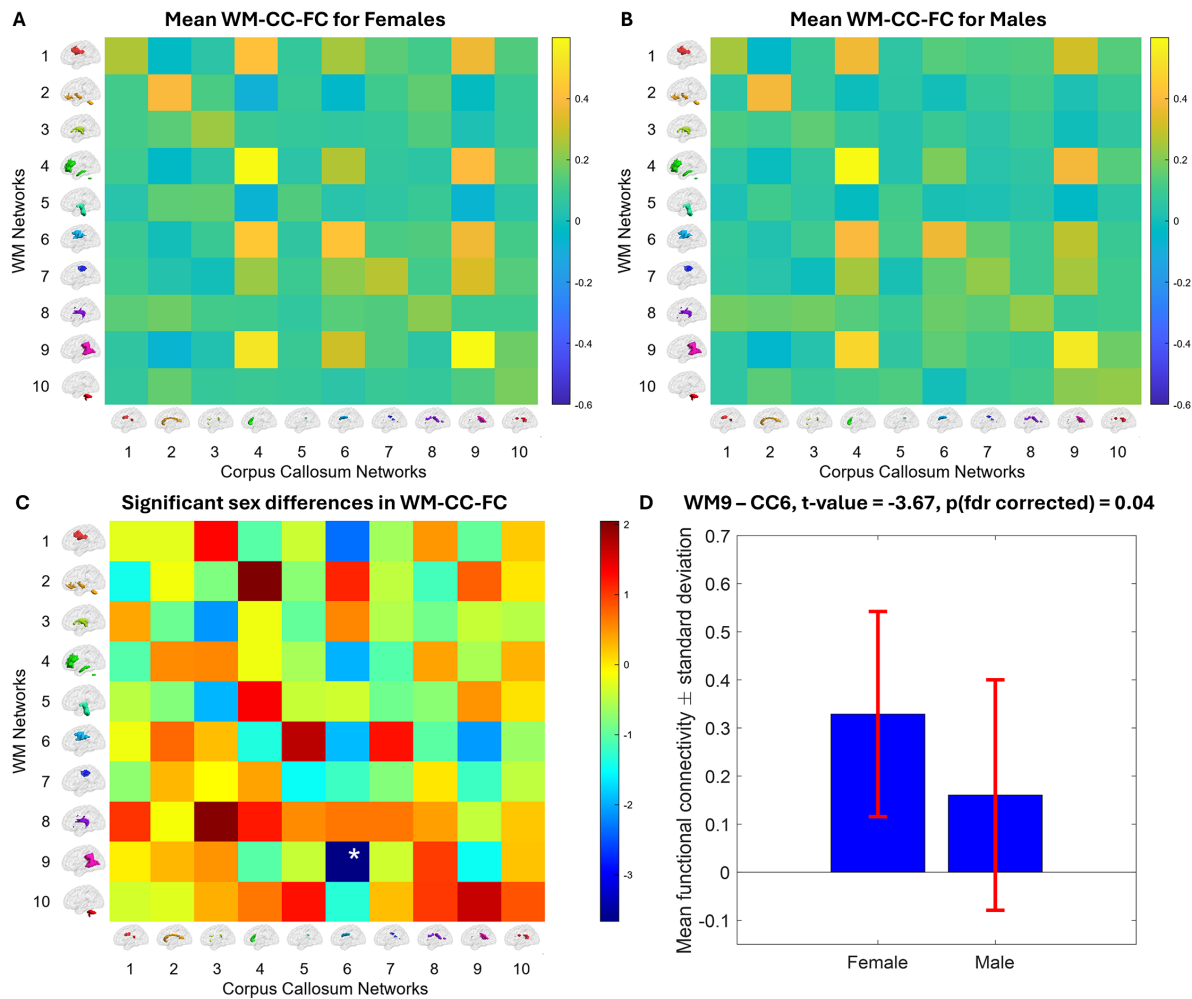
Sex differences in WM-CC-FC. Using the*Display FC*module, the mean WM-CC FC computed (A) across all females and (B) across all males was observed. (C) The t-values corresponding to different connections between males and females are shown. The significantly different connection is shown by *. FDR correction was used to correct for multiple comparisons. (D) The mean±std of Pearson correlation values between WM9 and CC6 across males and females obtained by the*bar plot*button in the*Statistics*module of WhiFuN. The connectivity between WM9 and CC6 was significantly higher in females than in males (t = 3.68, p(FDR corrected) = 0.04). Figures generated using the WhiFuN toolbox.

We found the connectivity between WM9 and CC6 to be significantly different between males and females (t = 3.67, p(FDR corrected) = 0.04),Figure 9. It can be observed fromFigure 9Ato C that the mean FC between WM9 and CC6 in females is 0.38, while the mean of the same connection in males is 0.15. The FNs created using the first session data were used to compute the FC for the second session (rest2). We found connectivity between WM9 and CC6 to also be significantly different between sexes when the second session data were used (t = 3.59, p(FDR corrected) = 0.026) (seeSupplementary Fig. S9).

To further validate the results, we created JHU-WM-FNs by combining the regions in the JHU-DTI-81 atlas that had the maximum dice coefficient with the corresponding WM-FN created by WhiFuN. The average time series was extracted from every JHU-WM-FN, and an FC matrix was created by correlating every average time series of the JHU-WM-FN with that of the CC atlas created by WhiFuN for every participant. By repeating the statistical analysis on JHU-WM-FNs, it was observed that the same network connection, JHU-WM9, with CC6, was significantly different (t = 3.97, p(FDR corrected) = 0.01) between the sexes which were also reproducible with the second session (t = 3.72, p(FDR corrected) = 0.03) (Supplementary Fig. S11).

Significant sex differences were also found in the WM-GM-FC, particularly in the connections of WM9-GM6 and WM7-GM8 (seeSupplementary Fig. S10); however, these were not reproducible using the second session data (rest2).

## 4 Discussion

Here, we present WhiFuN, an advanced neuroimaging toolbox, to analyze the BOLD signal from the WM regions of the human brain. This GUI-based toolbox offers researchers a user-friendly suite of automated tools for investigating brain functional connectivity in WM and GM. One of the key advantages of WhiFuN is that it fully automates the preprocessing steps to derive data that can be used to analyze the WM and GM BOLD signals. WhiFuN can be used to preprocess and analyzed rsfMRI and naturalistic stimuli paradigms. WhiFuN can aid in identifying differences in WM-FC across different cohorts. Users can easily create FNs, analyze FC, or use predefined atlas-FC maps to conduct group-level statistical analyses. The toolbox streamlines the complete workflow of analyzing WM and GM BOLD signals from preprocessing and quality control mechanisms, generating publication-ready visualizations of FNs and FC matrices, thereby accelerating WM BOLD analyses.

Preprocessing resting-state images can take a significant amount of time, depending on the spatial and temporal resolutions of the images. A completely automated preprocessing pipeline will help the user, as recurrent user interventions are unnecessary. The toolbox has robust error-handling capabilities, and errors do not interrupt the preprocessing pipeline. When an error occurs, WhiFuN will notify the user, discard that participant from further preprocessing, store the error information in the output folder, and continue preprocessing the remaining participants. After preprocessing is completed, the user can examine the error information saved by WhiFuN, fix the error, and run preprocessing on the skipped participant. WhiFuN automatically skips previously completed preprocessing steps and continues from the point of interruption. There can be a case where the computer unexpectedly shuts down while WhiFuN is preprocessing the participants. As all the preprocessing parameters are stored as a*.mat*file in the output folder, the user may load the parameters and rerun the preprocessing.

Another essential feature of WhiFuN is its robust visualization capabilities. WhiFuN automatically generates WM, GM, and CC FN maps with six different views as publication-quality image files that the user can use directly in manuscripts. These brain maps appear adjacent to the FC matrix (seeFig. 9A–C) to better visualize the connections. Moreover, the figures consisting of the FC matrices generated in the*Display FC*module and the group differences observed in the statistics module (seeFig. 9) can be generated by WhiFuN. The versatile visualization modules within WhiFuN help users to efficiently communicate the WM-FN research without the burden of postprocessing or external Figure generation.

Emphasis is given to separate GM and WM BOLD signals during preprocessing steps. This is crucial as the GM signals should not influence the WM-FNs (Peer et al., 2017). WhiFuN employs separate smoothing procedures for the GM and WM signals, relying on the tissue probability maps generated by SPM segmentation of anatomical images. We observed that any artifacts in the anatomical image or poor contrast of the anatomical image can lead to poor quality of the tissue probability maps. The poor-quality tissue probability maps would make identifying certain voxels in either the GM or WM difficult, and data from voxels having poor-quality tissue maps would be discarded during smoothing. This can impact WM and GM group masks, which consequently impact the FNs; thus, we recommend checking the quality of the anatomical images. The quality control plots for segmentation showing the voxels identified as GM, WM, and CSF should be observed carefully, and any misclassification of the tissue type should be manually identified. Participants with inadequate anatomical image quality, as determined by visual inspection, should be excluded. Readers can check what a GM and WM separately smoothed image looks like inSupplementary Figure S4. In particular, readers can observe voxels with no data as they were classified as CSF voxels, especially in the cortex. We plan to mitigate this problem by incorporating surface-based analysis for the GM and volume-based analysis for the WM in future toolbox versions.

The default threshold for the group WM mask is carefully chosen to avoid mixing GM signals with WM. We chose a threshold of 100% for the WM group mask compared to the 60% threshold suggested byPeer et al. (2017). Our data showed that if a threshold of 60% is used, GM voxels of at least one participant are included for about 39% of voxels in the WM mask. However, in their study, creating the WM-FNsPeer et al. (2017)excluded the voxels identified as GM at a subsequent participant level to create the average WM-FC matrix that was given as input to the K-Means algorithm. This step ensured that the GM voxels did not influence the WM-FNs. However, this creates slightly different WM-FNs for every participant, which affects the number of voxels used to compute the average time series for every FN across participants. Moreover, this FN cannot be directly used for any new participant. Furthermore, when we investigated sex differences in the FC created by the thresholds recommended byPeer et al. (2017), findings were not reproducible with the second session of fMRI. For the GM group mask, we use the same 20% threshold recommended byPeer et al. (2017). We found that most cortical areas would be discarded if a >20% GM group threshold was applied. This is due to the high spatial variance of the gyri and sulci between participants, which contributes to the GM probability of a particular voxel not being the same across participants.

For WM-CC FC, we found a connection between WM9 (which included left and right posterior corona radiata and posterior thalamic region) and the left body of the CC to be significantly different between males and females. The observed sex differences in WM FC align with prior findings on structural differences in the CC. Previous studies have reported sex-based variations in CC morphology, with females generally exhibiting a larger CC than males in both adults (Ardekani et al., 2013), and children (L. S. Allen et al., 1991). A larger CC might facilitate stronger connections and thus could explain why females have significantly higher FC as compared to males (seeFig. 9D). Additionally,Schmied et al. (2020)reported that the growth rate of CC in male infants was higher than that in female infants from ages 6 to 24 months. This suggests early developmental differences that could set the stage for later FC variations.Menzler et al. (2011)showed that men had higher fractional anisotropy (FA) and lower radial diffusivity in the thalamic and corpus callosum regions as compared to females. FA, a measure of WM integrity, could influence the efficiency of communication between the CC and sensory-related regions like the thalamus, contributing to FC differences. Significant sex differences were found in the WM engagement maps of the WM tracts, including the left Posterior Thalamic Radiation and Corpus Callosum. WM engagement measures how much each WM voxel contributes to GM-FC by totaling the FC values of all GM region pairs linked through that WM voxel. WM engagement maps were generated by first identifying all pairs of GM regions structurally connected through each WM voxel, as determined from DTI. Each WM voxel’s engagement in brain networks was quantified by summing the FC values of these GM pairs (from rsfMRI) weighted using a graph-theoretic approach (M. Li, Gao, et al., 2020). Finally, the findings of this paper were reproducible when analyzing FC from a second session of scans acquired from the same participants (rest2 of HCP), further supporting the robustness of this effect in the observed regions. While the exact mechanisms underlying these functional differences require further investigation, they could potentially be attributed to differences in callosal myelination, axonal organization, or interhemispheric communication efficiency between males and females.

WM and the GM-FNs were created independently without prior information on the hemispheres to which they belonged. Yet, K-means formed FNs that show symmetry across the hemispheres solely based on FC features. The GM-FNs computed by WhiFuN were mostly symmetric, similar to the widely used Yeo FNs (Yeo et al., 2011) (seeFig. 8A). The symmetry in the WM-FNs was first observed byPeer et al. (2017). However, in this study, we observed that in addition to some WM-FNs being symmetric, there were pairs of WM-FNs that were contra-lateral to each other (seeFig. 7A), as also observed using the ICA algorithm (Huang et al., 2020). Symmetry analysis can be used to examine the degree to which WM-FNs exhibit interhemispheric consistency. Given that the CC is the primary structure enabling interhemispheric communication, analyzing symmetry in WM-FNs may provide insights into how information transfer occurs between hemispheres. Deviations from expected symmetry patterns could be relevant in the study of neurodevelopmental or neurological disorders where interhemispheric communication is disrupted. For instance, the symmetric WM-FNs corresponding to the GM perception-motor system were altered in patients with schizophrenia, demonstrating that connections between the WM and GM-FNs are necessary to maintain the normal functionality of the brain (Jiang et al., 2019). Hemispheric communication between ipsilateral and contralateral WM FNs is also found to be disturbed in participants with temporal lobe epilepsy disorder (X. Li et al., 2022). Their findings highlight the importance of assessing symmetry in WM FC to understand its functional role in brain organization better. WhiFuN builds on this prior work by incorporating symmetry evaluation as a key analysis feature. Refer toSupplementary Figure S8, which uses the check FN symmetry module to quantify the symmetricity of the WM and GM FNs. Our current implementation of interhemispheric symmetry is applied only at the FN level. We aim to calculate the symmetry at the voxel level in future versions of WhiFuN.

To further validate and name the WM and GM-FNs, the FNs created by WhiFuN were compared with the widely used JHU-DTI-81 and Yeo FNs, respectively. Since the WM-FNs were compared with the JHU-DTI-81 atlas with regions smaller than the FNs, the dice coefficients were smaller than those of when the GM-FNs were compared with Yeo Networks. The WM-FNs created by WhiFuN had dice coefficients in the range of 0.1 to 0.5 compared to the JHU-DTI-81 atlas. WM2 FN had dice coefficients less than 0.1 for every region in the JHU atlas because it had regions in the cerebellum, right and left parts of deep cerebral WM, and left and right parts of forceps minor, which were not included in the JHU-DTI-81 atlas. However, this FN was symmetric, showing biological significance. The GM-FNs created using WhiFuN have a dice coefficient of 0.4 or higher with the cortical 7 Yeo FNs except for the GM4 FN, which has a dice coefficient of 0.35 with the Yeo limbic network (LN) and, interestingly, a 0.28 dice coefficient with the default mode network (DMN). This was due to a region named Inferior frontal Gyrus (IFG), which was found to be connected to the limbic areas of GM4 created by WhiFuN, but in the Yeo FNs, IFG is found in the DMN network. GM7 and GM9 have either sub-cortical regions or cerebellum regions. Yeo FNs only consider the cortical areas; hence, the dice coefficient of GM7 and GM9 was approximately 0 with all the Yeo FNs. The K-means algorithm independently identified all the prominent resting-state FNs without prior information, which shows that it can extract meaningful FNs.

Although the toolbox labels every voxel in the WM as belonging to one of the WM-FNs, it cannot further subdivide the FNs into parcellations or regions of interest, which can aid in seed-based FC analysis. Once the WM parcellations are created and the WM seed-based FC matrix is computed using the Pearson correlation, different graph theoretical measures, including global efficiency, node degree, density, and modularity, can be calculated using the brain connectivity toolbox and compared between two groups. Currently, this can only be achieved by using predefined atlases like the JHU atlas (included in the toolbox); however, this is a structural atlas created using DTI imaging. It would be interesting to create a functional WM atlas by dividing the brain regions based on FC. Furthermore, the stability of the FNs on an individual level is not robust; adding a spatial constraint or influencing the FNs with Bayesian analysis by having some prior information can improve the stability of the FNs at an individual level. These would help in finding individual differences and enhance brain-behavior correlation. The toolbox can only calculate FC, which gives no information on the causality. Effective Connectivity (EC) models the causal relations between brain regions (Stephan & Friston, 2010). EC in WM and between WM and GM can further elucidate the biophysical mechanics between WM and GM. We plan to include EC analysis in the future versions of the toolbox.

## Supplementary Material

Supplementary Material

## Data Availability

The HCP-unrelated 100 participants are openly available and free to download using connectome DB software fromhttps://www.humanconnectome.org. The toolbox can be downloaded fromhttps://github.com/Brain-Connectivity-Lab/WhiFuN
