# Supplementary material for: WhiFuN: A toolbox to map the white matter functional networks of the human brain
Source: Imaging Neurosci (Camb). 2025 May 30;3:IMAG.a.3. doi: 10.1162/IMAG.a.3 (PMC12319995; doi:10.1162/IMAG.a.3)
Supplement: Supplementary Material [file imag.a.3_supp.pdf]

WhiFuN supplementary document

Initial Check Plots

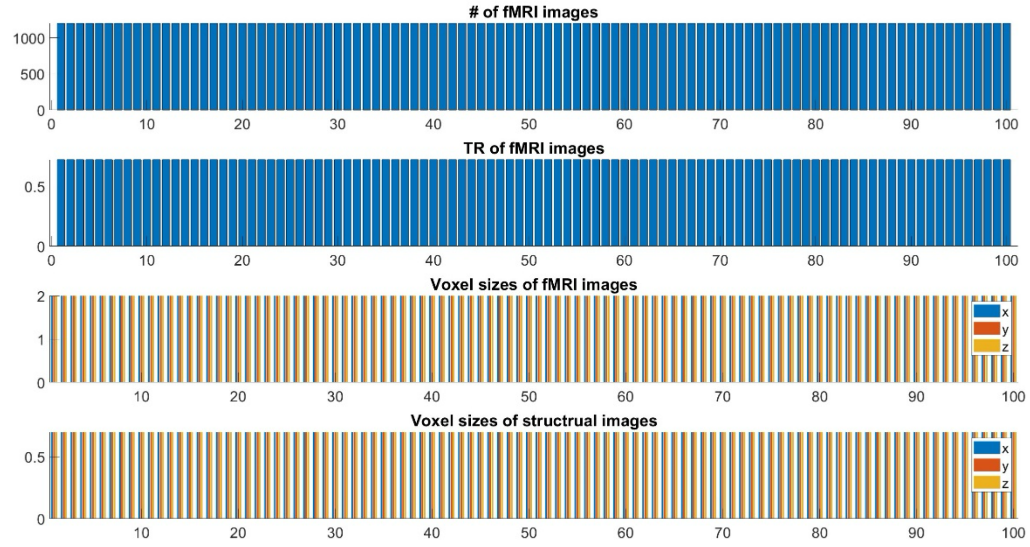

Figure S1:Bar plots of image parameters for every participant.

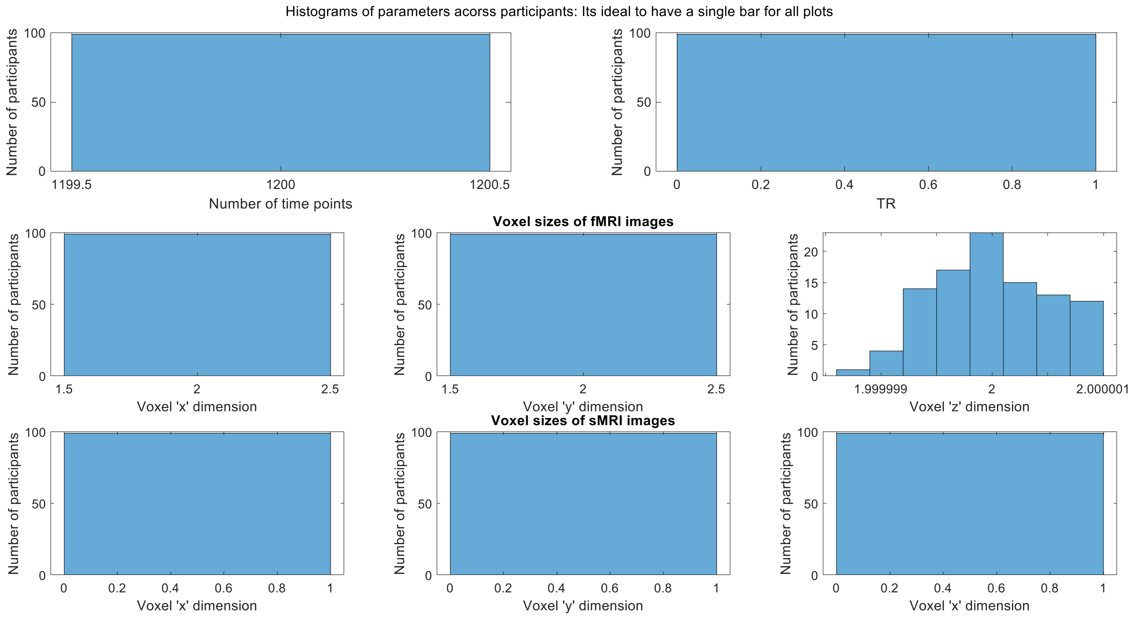

Figure S2: Histogram of the image parameters. Ideally, all participants should have the same parameters, hence there should only be 1 histogram bar for every parameter. Due to precision, the z-dimension size for the fMRI images has multiple bars from 1.99 to 2.01; this is acceptable but should be known.

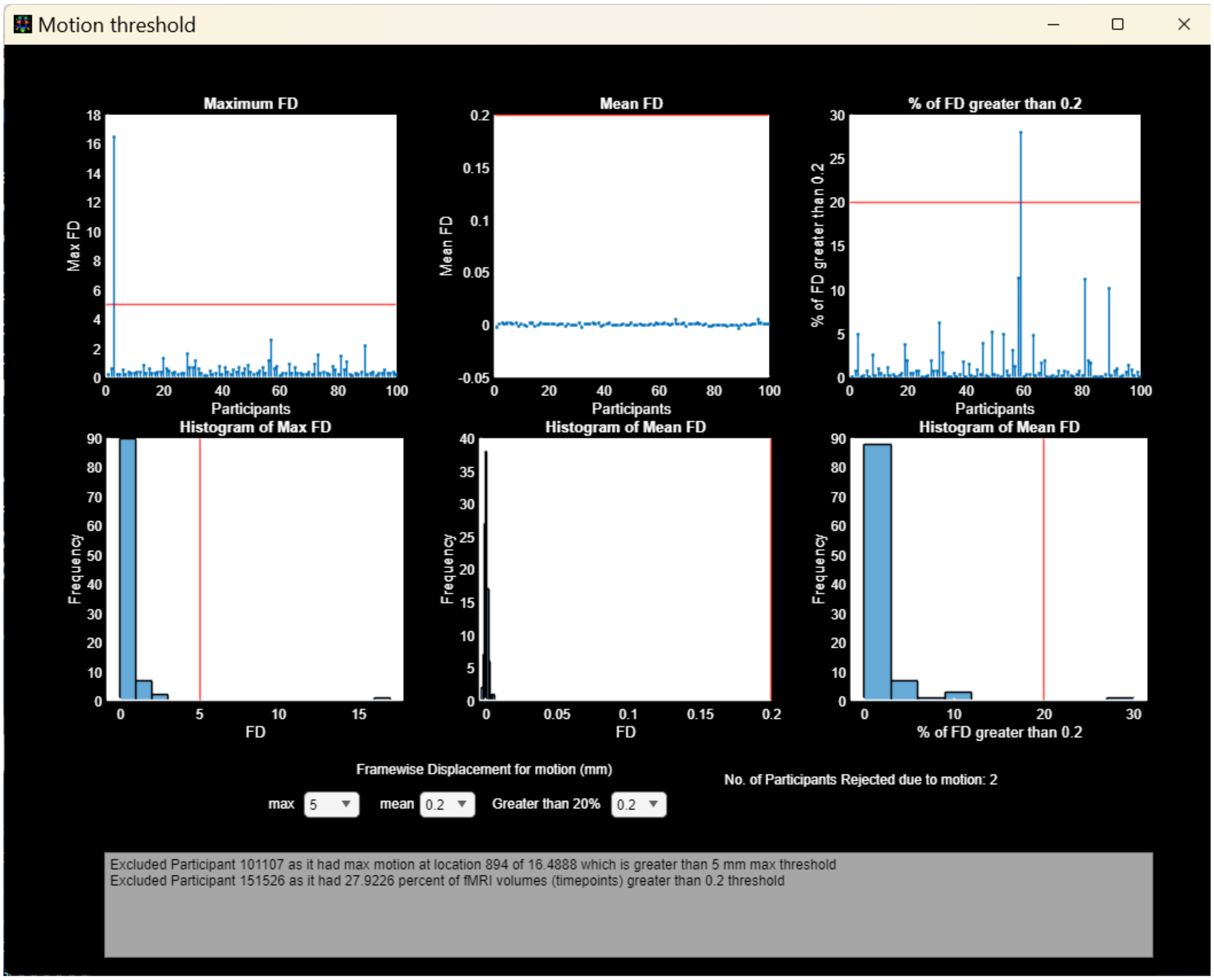

Figure S3: Determine Motion Thresholds module. Here the user can determine the Framewise displacement thresholds based on how many participants the user can afford to exclude and how much motion can be tolerated.

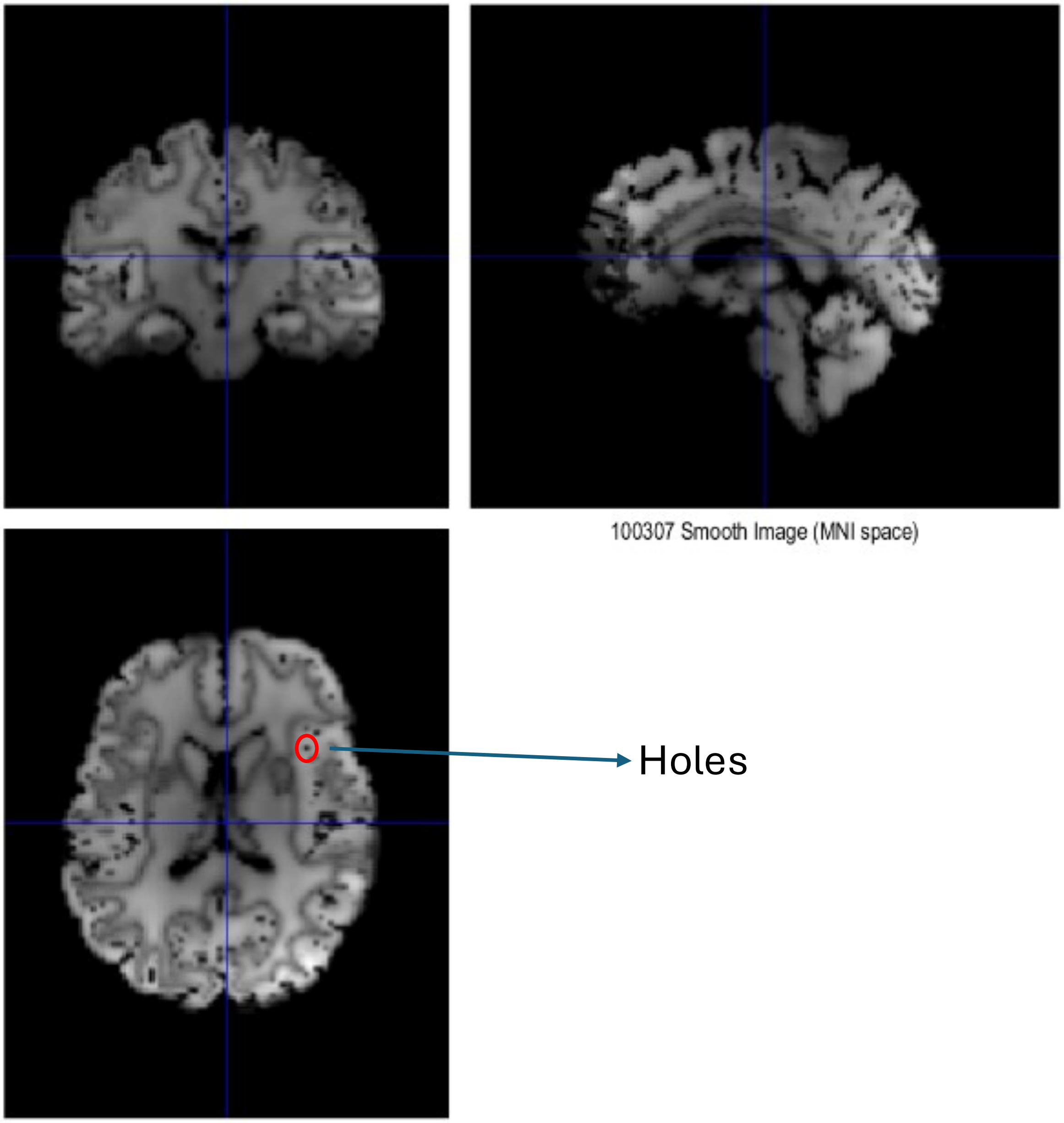

Figure S4: Smoothing GM and WM separately. The quality control plot saved after the smoothing WM and GM region separately. One can observe that there are a lot of holes where the data is not present. The voxels corresponding to these holes were classified as CSF voxels and hence were discarded. Here the classification of voxels as WM GM or CSF is done using the segmentation of the anatomical .Thus the contrast and quality of the anatomical image is very important at this step as a bad anatomical image will make it challenging to identify the tissue type of the voxels and thus make it difficult to smooth the WM and GM of rsfMRI image separately.

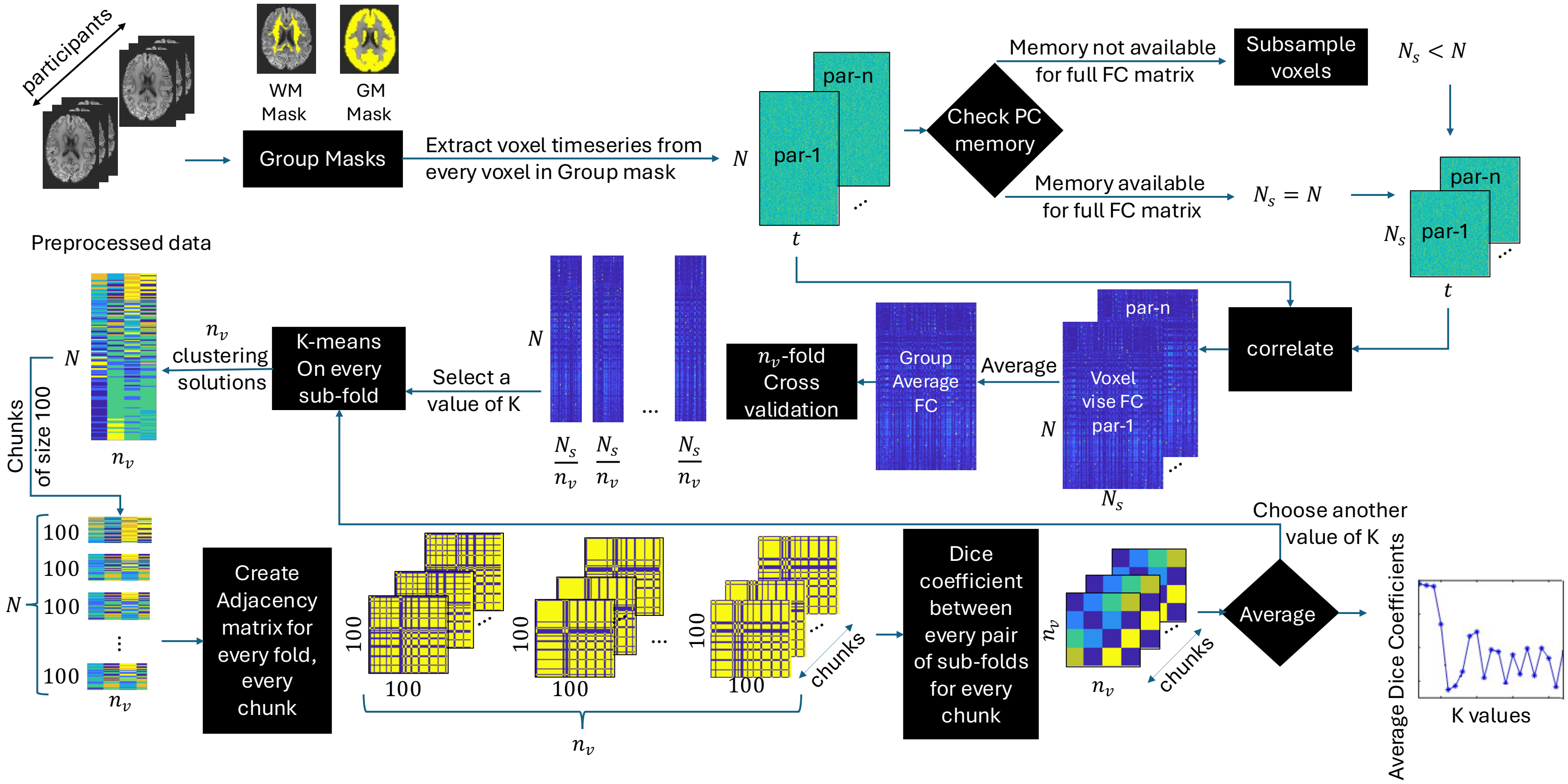

Figure S5: Computation of the Average Dice coefficient for every value of K (par → participant).

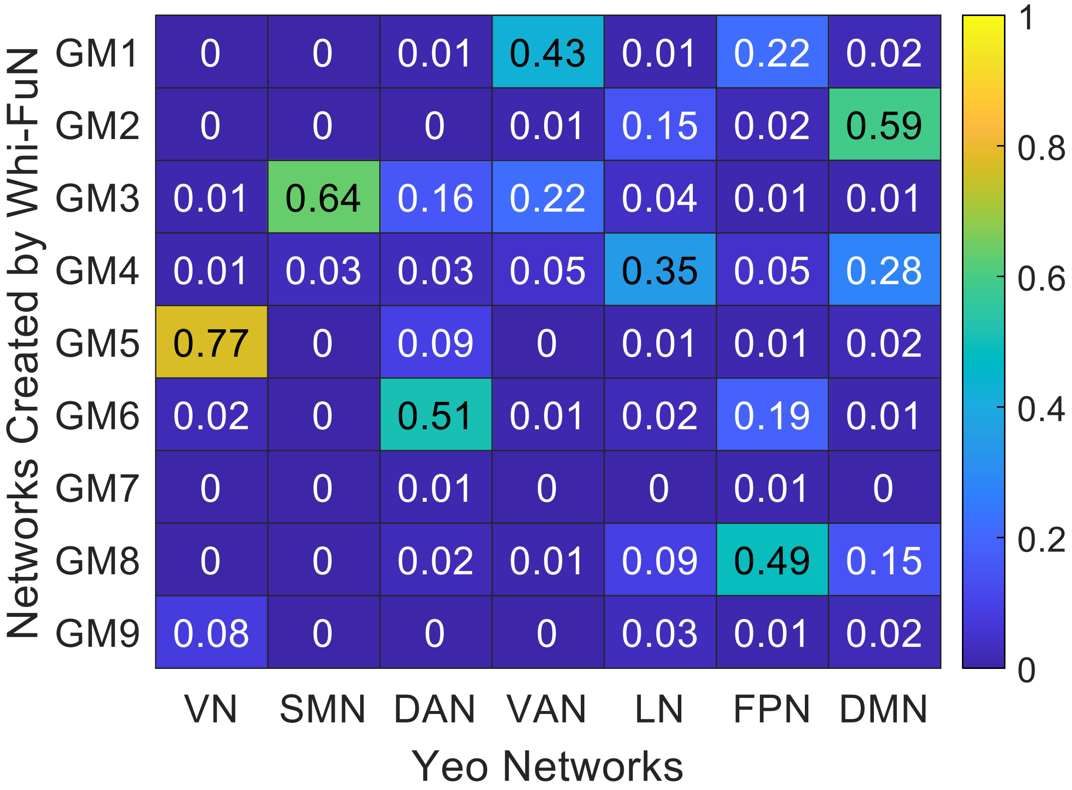

Figure S6: Comparing the GM-FNs created with WhiFuN to the 7 Yeo resting state Networks.

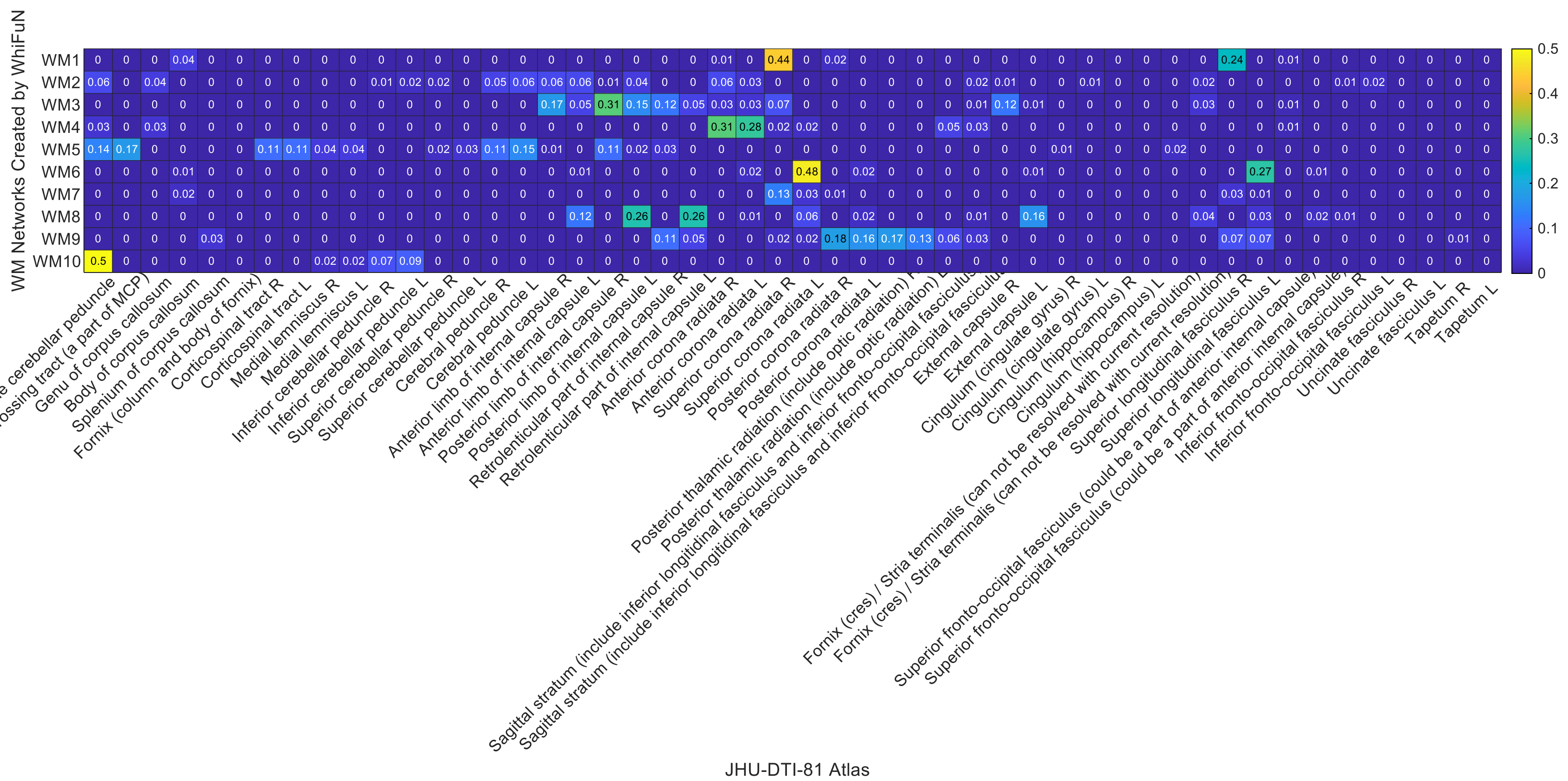

Figure S7: Comparing the WM-FNs created with WhiFuN to the JHU-DTI-81 Atlas.

Symmetricity of the networks between the left and right hemispheres

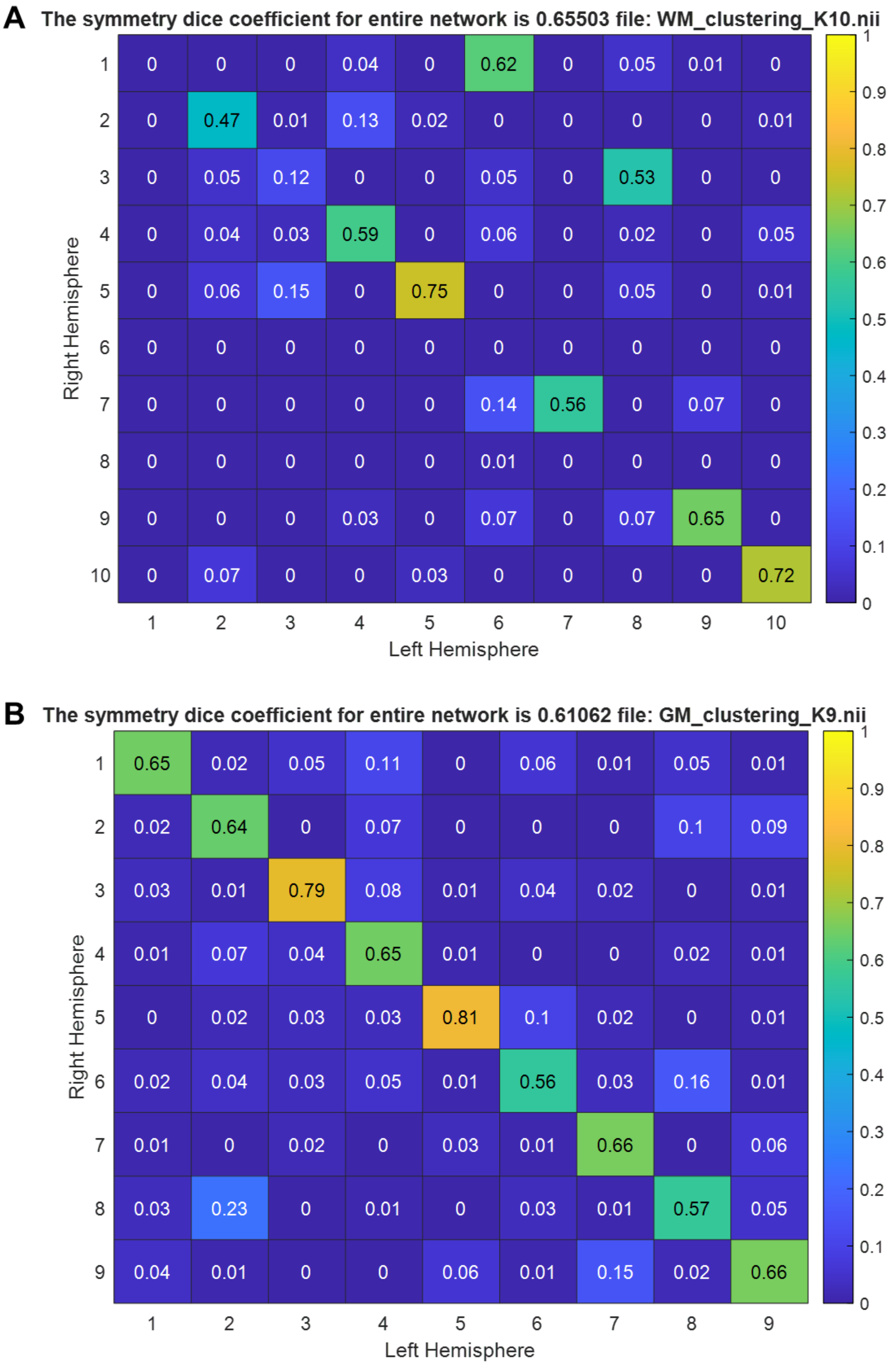

Figure S8: Dice coefficients computed between the FNs that are in the right hemisphere and FNs that are in the left hemisphere. A) Symmetry in WM-FNs. The WM-FNs are symmetric with a dice coefficient above 0.4 (see the diagonals). However, WM1 and WM3 are not symmetric to themselves but symmetric to WM6 and WM8, respectively. B) Symmetry in GM-FNs. All the GM-FNs are symmetric to themselves (see diagonals) with dice coefficients greater than 0.5. Figure generated using the Whi-FuN toolbox.

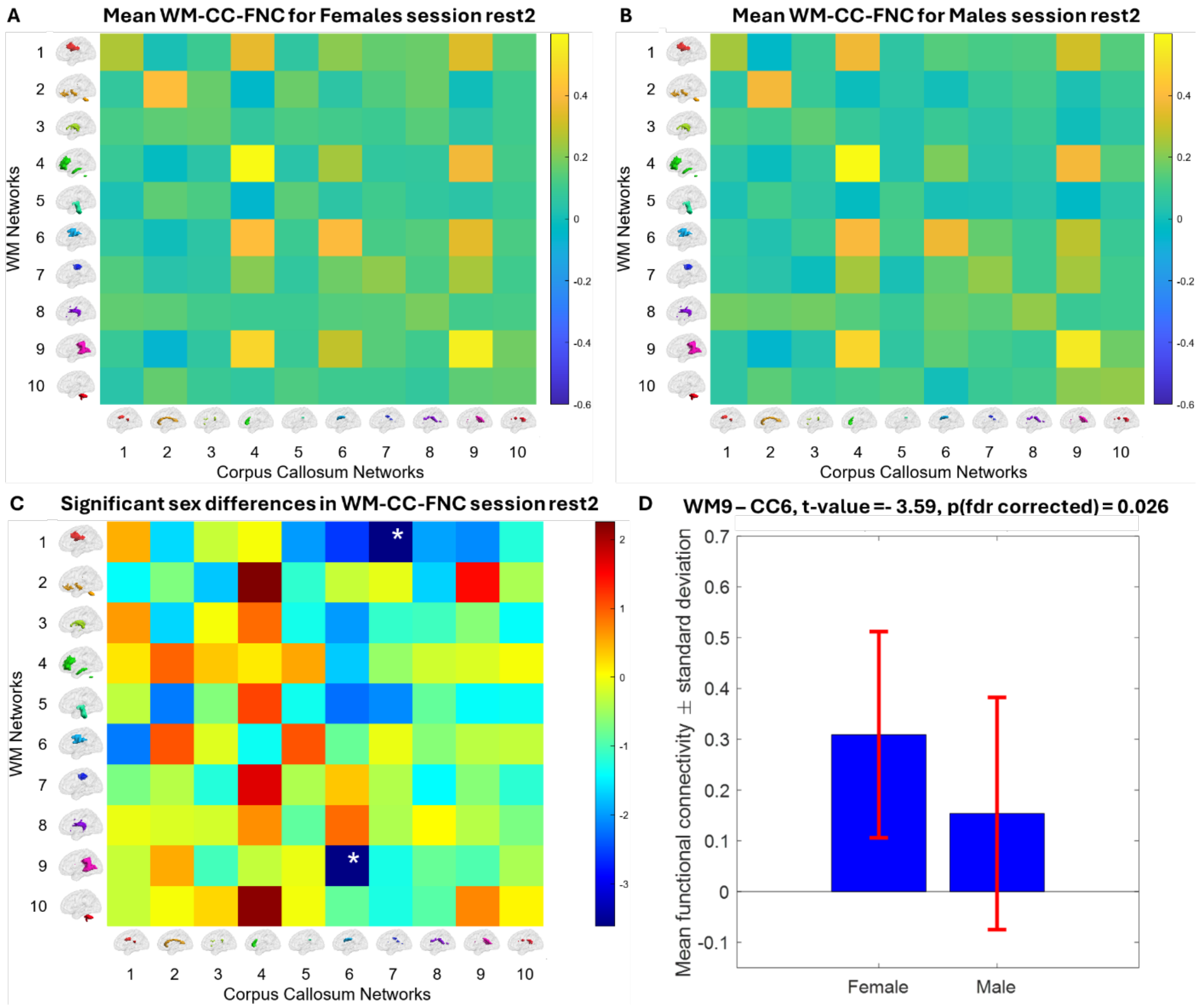

Figure S9: Sex differences in WM-CC FNC. Using the *FC/FNC visualize* module the mean WM-CC FNC computed **A:** across all females and **B:** across all males was observed. **C:** The t-values corresponding to different connections between males and females are shown. The significantly different connection is shown by *. FDR correction was used to correct for multiple comparisons. **D:** The mean ± std of Pearson correlation values between WM9 and CC6 across males and females obtained by the *bar plot* button in the *Statistics* module of WhiFuN. The connectivity between WM9 and CC6 was significantly higher in females than in males ($t = 3.59$, p (FDR corrected) = 0.026).

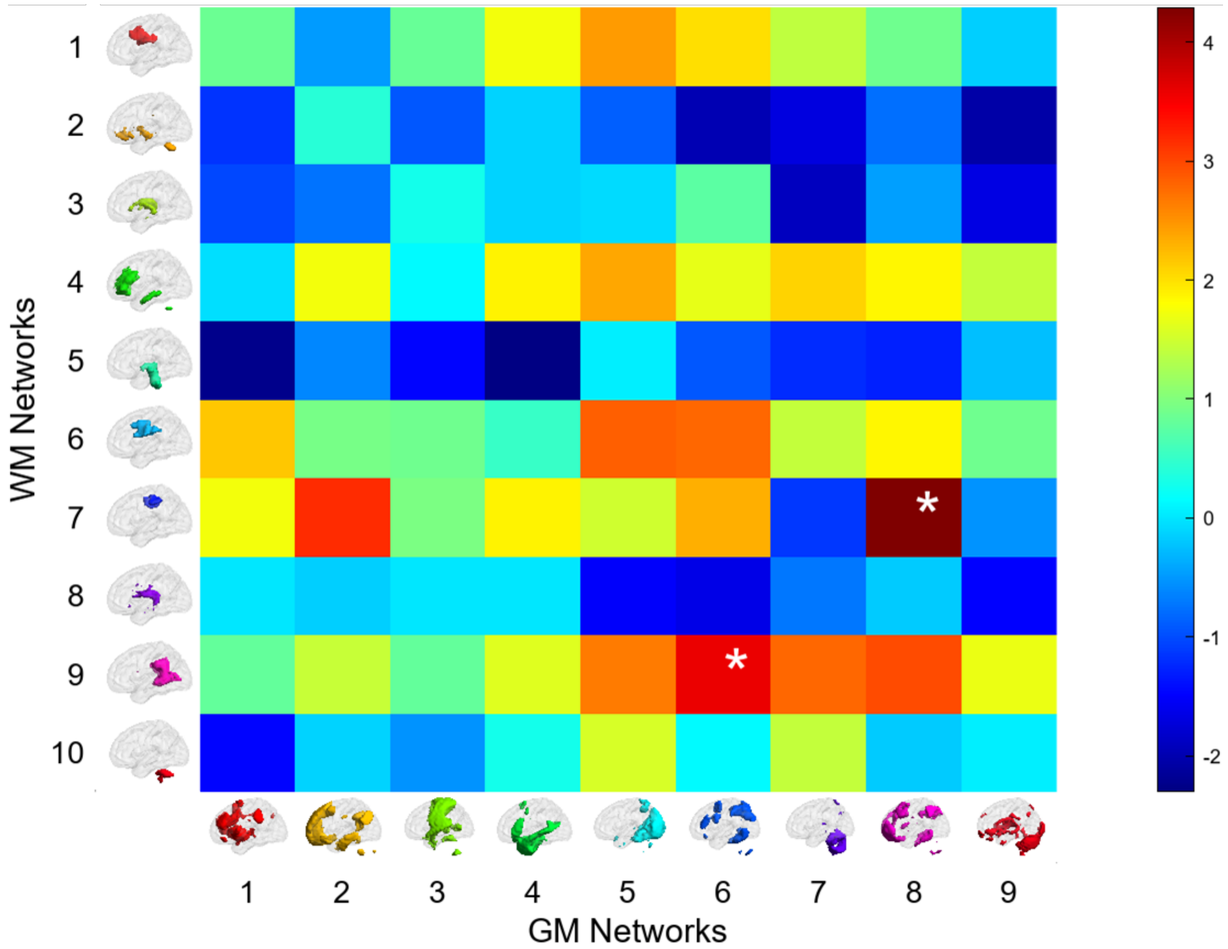

Figure S10: Sex differences in WM-GM FNC. Two connections were found to be significantly different between males and females in WM-GM-FNC (WM9-GM6, t=3.59, p(FDR corrected) = 0.02, WM7-GM8, two-sample t-test, t= 4.29, p(FDR corrected) = 0.0038). However, the second session data (rest 2-LR) did not reproduce these results.

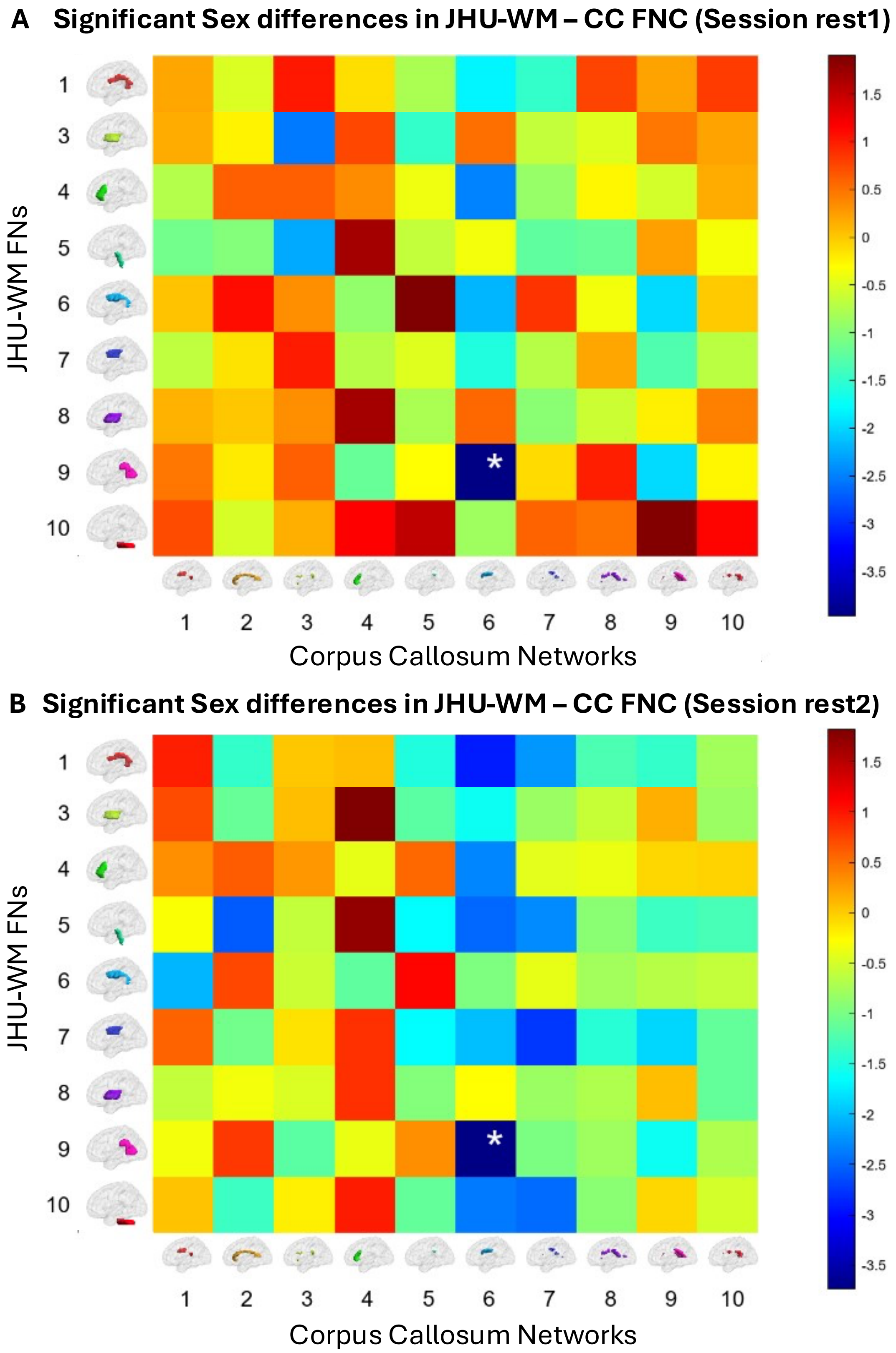

Figure S11: Sex differences in the JHU-WM-FNs created by combining the regions in the JHU-DTI 81 atlas that had maximum dice coefficient with the corresponding WM network created by Whi-

FuN. A) the t values computed using data from session 1 (rest1 LR). It can be observed that the same connection JHU-WM9 – CC6 has significant sex difference ($t=3.97$, p(FDR corrected) = 0.01) B) the t values computed using data from session 2 (rest2 LR). The results are reproducible using the 2nd session data as well ($t = 3.74$, p(FDR corrected) = 0.03)

JHU-WM-FNs are created by combining the JHU ROIs that have dice coefficients greater than 0.1 with the WM-FNs generated by WhiFuN. The table below shows the ROIs numbers in JHU-DTI-81 Atlas that were combined to create the corresponding JHU-WM-FNs.

Table : JHU-WM-FNs

JHU-WM-FN	JHU-DTI-81 ROIs
1	41
3	17, 19, 33
4	23, 24
5	2, 7, 8, 15, 16
6	26,42
7	25
8	18, 20, 34
9	27, 28, 29, 30
10	1
